# Machine learning methods for the detection and prediction of cognitive impairment in Parkinson’s disease: a systematic review and meta-analysis

**DOI:** 10.3389/fnagi.2025.1704039

**Published:** 2025-12-11

**Authors:** Hong Jiang, Xinling Yang, Wenxing Wang, Lin Jiang, Xiao’e Jiang

**Affiliations:** 1 The Second Affiliated Hospital, Xinjiang Medical University, Urumqi, China; 2 School of Nursing, Xinjiang Medical University, Urumqi, China; 3 Xinjiang Medical University, Urumqi, China; 4 The Sixth Affiliated Hospital of Xinjiang Medical University, Urumqi, China; 5 Department of Emergency, The First Affiliated Hospital of Xinjiang Medical University, Urumqi, China

**Keywords:** Parkinson’s disease, cognitive dysfunction, meta-analysis, calculation and diagnostic accuracy, systematic review, machine learning

## Abstract

**Background:**

Cognitive impairment in Parkinson’s disease (PD-CI) is a prevalent non-motor symptom, significantly diminishing quality of life and imposing a substantial family burden. Effective predictive tools are currently scarce, and the diagnostic pathway is intricate. With the growing use of artificial intelligence in healthcare, machine learning (ML) methodologies have been explored for the diagnosis and early risk prediction of PD-CI; however, their efficacy and accuracy necessitate systematic evaluation. Consequently, this investigation undertook a systematic review and meta-analysis.

**Method:**

A comprehensive literature retrieval was conducted across Web of Science, PubMed, Embase, and Cochrane Library, encompassing studies published from database inception to August 10, 2025. The PROBAST tool facilitated quality appraisal, ultimately incorporating 52 publications, of which 25 addressed diagnosis and 27 focused on risk prediction.

**Results:**

Findings indicated that within the validation cohorts, ML models for PD-CI diagnosis achieved a c-index of 0.82, with a sensitivity of 0.57 and specificity of 0.77. For PD-CI risk prediction, the c-index reached 0.83, accompanied by a sensitivity of 0.77 and specificity of 0.76. These results suggest that ML exhibits considerable accuracy in both the diagnosis and risk prediction of PD-CI. The models primarily incorporated variables such as clinical data, genetic characteristics, biomarkers, neuroimaging, and radiomics, and no overt signs of overfitting were detected.

**Conclusion:**

This research provides an evidence-based foundation for the future development of PD-CI risk prediction and intelligent diagnostic tools, thereby promoting the advancement and application of ML within Parkinson’s disease and related domains.

**Systematic review registration:**

https://www.crd.york.ac.uk/PROSPERO/, ID: CRD42023453586.

## 1 Introduction

Parkinson’s Disease (PD) stands as the second most prevalent extrapyramidal disorder and the second most frequently occurring degenerative disease of the central nervous system, second only to Alzheimer’s Disease (AD) (Pringsheim et al., 2014). A recent systematic review indicates that PD exhibits the most rapid increase in prevalence, disability, and mortality among neurological diseases (GBD 2016 Neurology Collaborators, 2019). Currently, there are approximately 7 million individuals diagnosed with PD worldwide, resulting in 211,296 fatalities. Notably, 10% of these patients are diagnosed before the age of 50, with incidence and prevalence rates increasing proportionally with age (Nemade et al., 2021; Valasaki, 2023). This significantly impacts the quality of life and social functioning of PD patients, potentially leading to disability. PD-associated cognitive impairment (PD-CI), as one of the non-motor symptoms in PD patients, is the most common and harmful syndrome (Goldman and Sieg, 2020). Currently, PD-CI diagnosis relies on criteria established by the International Parkinson and Movement Disorder Society (MDS) in 2015 (Berg et al., 2015). Research indicates that about 42% of PD individuals can develop PD-CI in the early stage of the disease (Aarsland et al., 2003); 20–57% can develop PD-mild cognitive impairment (PD-MCI) after 3–5 years; 78.2% of patients can progress to dementia after 8 years, and 20% ~ 42% of patients already had PD-CI at the time of consultation (Janvin et al., 2006; Agosta et al., 2014). In addition, studies have shown that 22–25% of PD-MCI patients can recover to PD with normal cognition (PD-NC), indicating that PD-MCI is reversible (Agosta et al., 2014; Brandão et al., 2020). Consequently, identifying suitable methods for early and accurate prediction, identification, and diagnosis of PD-CI holds significant potential for enhancing patient quality of life and alleviating familial and societal burdens.

The diagnosis of PD-MCI relies on limited support, exclusion criteria, and evaluation scales. However, this method is constrained by several factors, such as the interference of motor symptoms, patients’ lack of cooperation, the time-intensive nature of the examination, its labor-intensive nature, and the rich experience required from clinical experts. Consequently, it fails to provide accurate and objective predictions. Early warning and identification of PD-CI predominantly depend on clinical data, neuroimaging, neuroelectrophysiology, and radiomics. Nonetheless, these methods are limited by individual differences, inherent flaws in detection techniques, and complex and costly detection processes. These limitations pose significant challenges to both clinical professionals and family caregivers of PD patients (Kubota et al., 2016; Mostile et al., 2023). Consequently, developing intelligent and user-friendly predictive and diagnostic tools for early and precise identification of PD-CI, as well as prediction of its risk factors, holds significant clinical implications for the development of personalized prevention and treatment strategies, as well as the prediction of the prognosis of patients with PD-CI.

In recent years, machine learning (ML) has evolved alongside big data, offering significant advantages in complex data mining and analysis. Through data collection, processing, and computer algorithms, data is transformed into intelligent behavior. ML demonstrates high sensitivity, the ability to mine high-dimensional information, and high-throughput computing capacity. It facilitates the integration of intelligence with medical treatment and experimentation, which can assist in early risk prediction and differential diagnosis of diseases (Arumugam et al., 2023; Bhatt et al., 2023). Some studies have explored artificial intelligence-based methods to diagnose and predict the risk of PD-CI. However, the predictive and diagnostic performance of ML is still controversial due to the diverse methods and modeling variables. Therefore, our meta-analysis described the diagnostic and predictive accuracy of ML methods for PD-CI, which provided an evidence-based reference for future early detection and intelligent diagnostic tool development.

## 2 Methods

### 2.1 Study registration

Following the Preferred Reporting Items for Systematic Reviews and Meta-Analyses (PRISMA 2020) guidelines (Delgado-Alvarado et al., 2016; Moher et al., 2015), the current meta-analysis was implemented. Supplementary material 1 provides details of the guidelines. The study protocol was registered at the International Prospective Register of Systematic Reviews (PROSPERO) website (ID: CRD42023453586). Since all analyses were carried out based on previously published studies, ethical approval or informed consent was unnecessary.

### 2.2 Eligibility criteria

#### 2.2.1 Inclusion criteria

(1) We included studies reported in English;
(2) Participants were clinically diagnosed with PD;
(3) Studies had to construct a diagnostic or predictive model for PD-CI;
(4) At present, a large number of studies on ML lack an independent validation set, and only k-fold cross-validation was performed. They were also incorporated in our meta-analysis.

#### 2.2.2 Exclusion criteria

(1) Meta-analysis, systematic reviews, reviews, case studies, conference abstracts, and expert opinions on guidelines or animal experimentation;
(2) Studies that only predicted predictive factors or differential factors and did not construct comprehensive ML models (MLMs) were excluded;
(3) Outcome measures of accuracy of diagnostic and predictive models were not reported, such as c-index, the receiver operating characteristic (ROC) curve, specificity, sensitivity, accuracy, precision, recovery rate, confusion matrix, F1 score, calibration curve, and diagnostic grid table;
(4) Conference summaries published without undergoing peer review were also excluded.

### 2.3 Literature search strategy

We searched the PubMed, Embase, Cochrane Library, and Web of Science databases up to August 10, 2025. The search strategy used a combination of subject terms and free-text words without restricting the year or geographical area. Supplementary material 2 presents the detailed strategies.

### 2.4 Study selection and information acquisition

Articles retrieved were imported into EndNote. Then, duplicates were manually and automatically identified and removed. The titles and abstracts of the remaining articles were checked. For potentially relevant articles, full texts were downloaded and read to identify eligible articles for this systematic review.

Prior to data extraction, we developed a standardized spreadsheet. The extracted information included the title, first author, study design, geographic location, year of publication, country, type of subjects, source of patients, diagnostic criteria for PD-CI, type of model, number of patients in the dataset, internal and external validation, processing method for overfitting, procedure for missing data, feature screening method, the total number of PD-MCI patients, model name, the total number of PD patients, predictor type, the total number of PD patients in the training dataset, predictive performance measures, the generation method of validation dataset, the treatment of missing data, the number of cases in the validation dataset, type of model, the number of PD-MCI cases in the validation dataset, modeling variables, the evaluation of overfitting, and the availability of code and data.

Two researchers (HJ and W-X W) screened literature and collected information separately, followed by mutual verification; discrepancies were settled through consultation with a third researcher (X-L Y).

### 2.5 Risk of bias in studies

All models in the training set were evaluated by two researchers (HJ and LJ) using the Prediction Model Risk of Bias Assessment Tool (PROBAST), followed by interactive inspection. Discrepancies were resolved through arbitration by a third researcher (X-L Y).

PROBAST is a tool designed to systematically evaluate the risk of bias in prognostic or diagnostic prediction models (Mukherjee et al., 2017). This assessment encompasses four key elements: participants, predictors, outcomes, and analyses. Each element was evaluated and categorized as “high,” “unclear” (indicating insufficient details or non-reporting), or “low” risk of bias based on the specific characteristics of the eligible studies. “High” signifies a higher risk of bias, while “Low” denotes a lower risk. An overall risk of bias was deemed to be low when all domains were significantly at a low risk. Conversely, if one or more domains displayed a significantly high risk, the overall risk of bias was considered high.

### 2.6 Outcomes

Our research incorporated both diagnostic and predictive models. The c-index was utilized as the primary outcome measure to assess the overall accuracy of MLMs. However, in instances where the number of cases was significantly unbalanced between the control and observation groups or the sample size was excessively small, the c-index was inadequate for assessing the precision of ML in the prediction and diagnosis of PD-CI. Consequently, specificity and sensitivity were included as primary measures. In the current ML landscape, most models output the probability of a positive outcome. ROC curves, plotted based on these probabilities, are a common method for evaluating overall model accuracy. Previous investigations often overlooked sensitivity and specificity, key metrics of a model’s core diagnostic ability for positive events at the optimal probability threshold. To address this oversight, the present study introduced the c-index as a crucial threshold indicator for precisely quantifying this core diagnostic capability.

### 2.7 Synthesis methods

The current meta-analysis examined the predictive and diagnostic accuracy of ML for PD-CI. In terms of the meta-analysis of predictive accuracy, PD patients did not develop cognitive impairment at baseline, and ML was used to forecast the risk of developing cognitive impairment in subsequent survival periods. In the meta-analysis of diagnostic accuracy, PD patients may develop cognitive impairment at baseline, and ML was used to pinpoint those with cognitive impairment.

We conducted a meta-analysis assessing the metric for evaluating the overall accuracy of MLMs, specifically the c-index. In instances where the 95% confidence interval and standard error of the c-index were unavailable in the original studies, we estimated the standard error by referencing the study conducted by Debray et al. (2019). Considering the disparities in included variables and inconsistent parameters among MLMs, our meta-analysis of the c-index prioritized the use of a random-effects model. Furthermore, we conducted a meta-analysis of sensitivity and specificity utilizing a bivariate mixed-effects model (Reitsma et al., 2005). During this meta-analysis, the diagnostic four-fold table was employed to analyze sensitivity and specificity; however, most original studies failed to report a four-fold table. In response, we adopted two methods for calculating the diagnostic four-fold table: (1) Combining sensitivity, specificity, and precision with the number of cases; (2) Extracting sensitivity and specificity based on the optimal Youden’s index, and then calculating sensitivity and specificity based on the number of cases. The meta-analysis was executed using R4.2.0.

## 3 Results

### 3.1 Study selection

**Figure 1:**
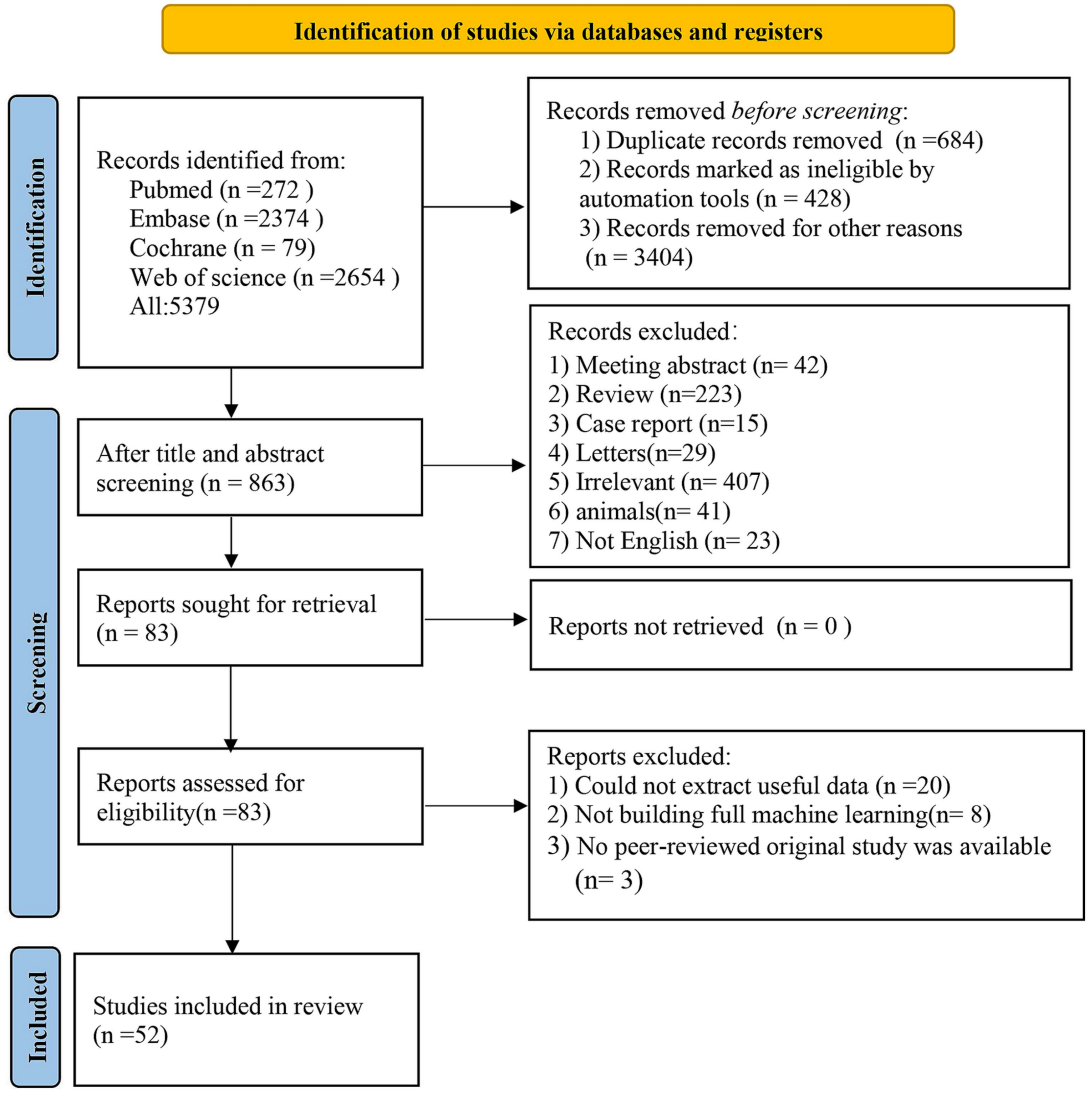
Literature screening process.

Database retrieval yielded 5,397 articles, 684 of which were removed as duplicates. Among the remaining 4,695 articles, 4,610 were excluded based on screening of the titles or abstracts, leaving 85 potentially relevant, original, English-language articles published in peer-reviewed journals. Full texts of these 83 articles were then evaluated for eligibility. Thirty-one articles were removed (including 20 due to due to unavailable data, eight that did not implement a complete ML framework, and three that were not original peer-reviewed studies). Ultimately, the meta-analysis incorporated 52 eligible articles (Liu et al., 2017; Schrag et al., 2017; Hogue et al., 2018; Zhang et al., 2021; Tang et al., 2021; Russo et al., 2023; Chen et al., 2022; Harvey et al., 2022; Zhang et al., 2022; Chen et al., 2023; Chen et al., 2021; Abós et al., 2017; Zhang et al., 2020; García et al., 2021; Ortelli et al., 2022; Amboni et al., 2022; Koch et al., 2019; Shen et al., 2022; Brien et al., 2023; Kang et al., 2022; Shibata et al., 2022; Chung et al., 2021; Chang et al., 2022; Novak et al., 2021; Gschwandtner et al., 2023; Cai et al., 2021; Hou et al., 2024; Park et al., 2023; Sivaranjini and Sujatha, 2024; Huang et al., 2024; Baek et al., 2024; Parajuli et al., 2023a; Liu et al., 2023; McFall et al., 2023; Zhang et al., 2023; Gorji and Fathi Jouzdani, 2024; Fiorenzato et al., 2024; Zhu et al., 2024; Beheshti and Ko, 2024; Hosseinzadeh et al., 2023; Jian et al., 2024; Li H. et al., 2025; Luo et al., 2025; d'Angremont et al., 2025; Li L. et al., 2025; Silva-Rodríguez et al., 2025; Beheshti et al., 2024; Putha et al., 2025; Kemp et al., 2025; Mostile et al., 2025; Parajuli et al., 2023b; Liu et al., 2025). Figure 1 illustrates the PRISMA flowchart outlining the study selection.

### 3.2 Study characteristics

The final set of 52 studies were published between 2017 and 2025, originating from Argentina (Pringsheim et al., 2014; García et al., 2021), Spain (GBD 2016 Neurology Collaborators, 2019; Abós et al., 2017; Silva-Rodríguez et al., 2025), Canada (Goldman and Sieg, 2020; Brien et al., 2023; McFall et al., 2023; Beheshti and Ko, 2024; Hosseinzadeh et al., 2023; Beheshti et al., 2024), China (Reitsma et al., 2005; Zhang et al., 2021; Tang et al., 2021; Zhang et al., 2022; Chen et al., 2023; Chen et al., 2021; Zhang et al., 2020; Kang et al., 2022; Chang et al., 2022; Cai et al., 2021; Hou et al., 2024; Huang et al., 2024; Liu et al., 2023; Zhang et al., 2023; Zhu et al., 2024; Jian et al., 2024; Li H. et al., 2025; Luo et al., 2025; Li L. et al., 2025; Liu et al., 2025), South Korea (GBD 2016 Neurology Collaborators, 2019; Park et al., 2023; Baek et al., 2024), India (Pringsheim et al., 2014; Sivaranjini and Sujatha, 2024), Iran (Pringsheim et al., 2014; Gorji and Fathi Jouzdani, 2024), Netherlands (Pringsheim et al., 2014; d'Angremont et al., 2025), Germany (Pringsheim et al., 2014; Koch et al., 2019), Japan (Pringsheim et al., 2014; Shibata et al., 2022), Switzerland (Pringsheim et al., 2014; Gschwandtner et al., 2023), USA (Janvin et al., 2006; Liu et al., 2017; Hogue et al., 2018; Shen et al., 2022; Novak et al., 2021; Parajuli et al., 2023a; Putha et al., 2025; Kemp et al., 2025; Parajuli et al., 2023b), Taiwan (GBD 2016 Neurology Collaborators, 2019; Chen et al., 2022; Chung et al., 2021), UK (GBD 2016 Neurology Collaborators, 2019; Schrag et al., 2017; Harvey et al., 2022), Italy (Goldman and Sieg, 2020; Russo et al., 2023; Ortelli et al., 2022; Amboni et al., 2022; Fiorenzato et al., 2024; Mostile et al., 2025). Of these, 27 studies (Liu et al., 2017; Schrag et al., 2017; Hogue et al., 2018; Tang et al., 2021; Harvey et al., 2022; Zhang et al., 2022; Chen et al., 2021; Shen et al., 2022; Brien et al., 2023; Gschwandtner et al., 2023; Hou et al., 2024; Park et al., 2023; Huang et al., 2024; McFall et al., 2023; Zhang et al., 2023; Gorji and Fathi Jouzdani, 2024; Zhu et al., 2024; Beheshti and Ko, 2024; Hosseinzadeh et al., 2023; Jian et al., 2024; Li H. et al., 2025; Luo et al., 2025; d'Angremont et al., 2025; Li L. et al., 2025; Beheshti et al., 2024; Putha et al., 2025; Mostile et al., 2025) focused on predicting the risk of PD-CI, while another 25 (Zhang et al., 2021; Russo et al., 2023; Chen et al., 2022; Chen et al., 2023; Abós et al., 2017; Zhang et al., 2020; García et al., 2021; Ortelli et al., 2022; Amboni et al., 2022; Kang et al., 2022; Shibata et al., 2022; Chung et al., 2021; Chang et al., 2022; Novak et al., 2021; Cai et al., 2021; Sivaranjini and Sujatha, 2024; Baek et al., 2024; Parajuli et al., 2023a; Liu et al., 2023; Fiorenzato et al., 2024; Luo et al., 2025; Silva-Rodríguez et al., 2025; Kemp et al., 2025; Parajuli et al., 2023b; Liu et al., 2025) aimed to diagnose the status of PD-CI. Fifteen studies (Zhang et al., 2022; Zhang et al., 2020; Koch et al., 2019; Kang et al., 2022; Chung et al., 2021; Chang et al., 2022; Hou et al., 2024; Park et al., 2023; Liu et al., 2023; Zhang et al., 2023; Zhu et al., 2024; Li H. et al., 2025; d'Angremont et al., 2025; Mostile et al., 2025; Liu et al., 2025) were single-center, and 26 (Liu et al., 2017; Schrag et al., 2017; Hogue et al., 2018; Tang et al., 2021; Harvey et al., 2022; Chen et al., 2021; Abós et al., 2017; Ortelli et al., 2022; Amboni et al., 2022; Shen et al., 2022; Shibata et al., 2022; Sivaranjini and Sujatha, 2024; Huang et al., 2024; Baek et al., 2024; Parajuli et al., 2023a; McFall et al., 2023; Gorji and Fathi Jouzdani, 2024; Fiorenzato et al., 2024; Beheshti and Ko, 2024; Hosseinzadeh et al., 2023; Jian et al., 2024; Luo et al., 2025; Beheshti et al., 2024; Putha et al., 2025; Kemp et al., 2025; Parajuli et al., 2023b) were multi-center. Eleven studies (Zhang et al., 2021; Russo et al., 2023; Chen et al., 2022; Chen et al., 2023; García et al., 2021; Brien et al., 2023; Novak et al., 2021; Gschwandtner et al., 2023; Cai et al., 2021; Li L. et al., 2025; Silva-Rodríguez et al., 2025) reported recruitment details. A total of 89 MLMs were identified, including support vector machines (SVM: 13), convolutional neural networks (CNN: 6), linear discriminant analysis (LDA: 8), logistic regression (LR: 17), artificial neural networks (ANN: 7), decision trees (DT: 2), random forest (RF: 12), linear mixed-effects models (LME: 9), Cox proportional hazards regression models (COX: 7), naïve Bayes (NB: 3), and extreme gradient boosting (XGBoost: 5).

The sample sizes ranged from 20 to 1,293 participants. Concerning PD-CI diagnosis, cognitive assessment was conducted via the Montreal Cognitive Assessment (MoCA) in 30 studies (Schrag et al., 2017; Hogue et al., 2018; Tang et al., 2021; Zhang et al., 2022; García et al., 2021; Shen et al., 2022; Brien et al., 2023; Kang et al., 2022; Shibata et al., 2022; Novak et al., 2021; Cai et al., 2021; Hou et al., 2024; Sivaranjini and Sujatha, 2024; Huang et al., 2024; Baek et al., 2024; Parajuli et al., 2023a; Liu et al., 2023; Gorji and Fathi Jouzdani, 2024; Zhu et al., 2024; Beheshti and Ko, 2024; Hosseinzadeh et al., 2023; Jian et al., 2024; Luo et al., 2025; d'Angremont et al., 2025; Li L. et al., 2025; Silva-Rodríguez et al., 2025; Beheshti et al., 2024; Putha et al., 2025; Kemp et al., 2025; Parajuli et al., 2023b); while the Minimum Mental State Examination (MMSE) was employed in five (Liu et al., 2017; Chen et al., 2022; Abós et al., 2017; Li H. et al., 2025; Parajuli et al., 2023b). Eight studies (Zhang et al., 2021; Chen et al., 2023; Zhang et al., 2020; Chung et al., 2021; Chang et al., 2022; Gschwandtner et al., 2023; Zhang et al., 2023; Fiorenzato et al., 2024) utilized either MoCA or MMSE for scoring. One study (Ortelli et al., 2022) applied the Cognitive Disorders in Movement Disorders Assessment (CoMDA) scale, and another (Koch et al., 2019) integrated the SENS-PD-COG, MMSE, and MoCA scales for cognitive evaluation. An additional study (Harvey et al., 2022) combined HVLT-R discrimination scores, MoCA, and SFT-Geget scores. Three studies (Russo et al., 2023; McFall et al., 2023; Mostile et al., 2025) based their assessments on DSM-5 criteria for dementia, and one (Amboni et al., 2022) applied the Movement Disorder Society-Unified Parkinson’s Disease Rating Scale (MDS-UPDRS). One study (Chen et al., 2021) followed the MDS diagnostic criteria for PD-MCI, and another (Ortelli et al., 2022) again utilized the CoMDA scale. Key modeling variables were selected based on a combination of clinical characteristics and magnetic resonance imaging (MRI), cerebrospinal fluid biomarkers, radiomics, and electroencephalography (EEG), all of which demonstrated significant utility in PD-CI diagnosis. The most frequently used predictors included neuroimaging data, radiomic features, genetic scores, MMSE, sex, MoCA, disease duration, age, and composite scale scores. The basic features of the eligible studies are presented in Table 1.

**Table 1:** Basic information of the eligible studies.

No.	References	Country	Types of models	Total sample size	Cases of cognitive impairment	Sample size in training set	Verification method	Sample size in validation set	Overfitting method	Type of model	Study design	Primary outcome	Diagnosis of cognitive impairment	Patient source
1	Liu et al. (2017)	USA	Prediction model	–	–	3,200	External validation	1,132	–	COX	cohort study	C-index	MMSE	Multicenter
2	Schrag et al. (2017)	UK	Prediction model	314	52	220	10-fold cross validation	94	10-fold cross validation	LR	cohort study	C-index	MoCA	Multicenter
3	Hogue et al. (2018)	USA	Prediction model	315	38	315	Bootstrap	315	Bootstrap	Logistical	cohort study	C-index	MoCA	Multicenter
4	Zhang et al. (2021)	China	Diagnostic model	71	36	56	5-fold cross validation	15	5-fold cross validation	SVM	case–control study	C-index	MoCA or MMSE	Recruit
5	Chung et al. (2021)	China	Prediction model	108	58	86	Random sampling	22	–	COX	cohort study	C-index	MoCA	Multicenter
6	Russo et al. (2023)	Italy	Diagnostic model	80	40	60	cross validation	20	cross validation	DT/RF/NB/SVM	case–control study	C-index, Sensitivity and specificity	DSM-V	Recruit
7	Chen et al. (2022)	Taiwan	Diagnostic model	42	26	30	Random sampling	12		SVM	cohort study	C-index, Sensitivity and specificity	MMSE	Recruit
8	Harvey et al. (2022)	UK	Prediction model	209	82	125	10-fold cross validation	84	10-fold cross validation	ElasticNet/RF/SVM	cohort study	C-index	HVLT-R + MoCA+SFT-Geget	Multicenter
9	Zhang et al. (2022)	China	Prediction model	405	205		Bootstrap	–	Bootstrap	logistic/LASSO regression	case–control study	C-index	MoCA	Single-center
10	Chen et al. (2023)	China	Diagnostic model	120	68	96	Random sampling	24	10-fold cross validation	DT/RF/XGBoost	case–control study	C-index, Sensitivity and specificity	MoCA or MMSE	Recruit
11	Chen et al. (2021)	China	Prediction model	338	62	96	Random sampling	24		Cox/LASSO	cohort study	C-index	MDS-PD-MCI	Multicenter
12	Abós et al. (2017)	Spain	Diagnostic model	95	35	70	leave-one-out cross-validation	25	10-fold cross validation	Logistic/RLR/SVM	case–control study	C-index, Sensitivity and specificity	MMSE	Multicenter
13	Zhang et al. (2020)	China	Diagnostic model	93	58	73	Random sampling 5-fold cross validation	20	Leave-one-out cross-validation	SVM	case–control study	C-index, Sensitivity and specificity	MoCA or MMSE	Single-center
14	García et al. (2021)	Argentina	Diagnostic model	80	40	93	5-fold cross validation	–	–	SVM	Case–control study	C-index, Sensitivity and specificity	MoCA	Recruit
15	Ortelli et al. (2022)	Italy	Diagnostic model	500		349	10-fold cross validation	151	Cross validation	CoMDA-ML	Cohort study	C-index, Sensitivity and specificity	CoMDA	Multicenter
16	Amboni et al. (2022)	Italy	Diagnostic model	75	33	75	10-fold cross validation	–	Leave-one-out cross-validation	KNN/SVM/RF/J48/ADA-B	Cohort study	C-index, Sensitivity and specificity	MDS-UPDRS	Multicenter
17	Koch et al. (2019)	Germany	Diagnostic model	40	20		10-fold cross validation	–	10-fold cross validation	RF	Case–control study	C-index, Sensitivity and specificity	SENS-PD-COG+MMSE+MoCA	Single-center
18	Shen et al., 2022	USA	Prediction model	191	52	108	External validation	83	5-fold cross validation	Cox	Cohort study	C-index	MoCA	Multicenter
19	Brien et al. (2023)	Canada	Prediction model	121	65	121	–			SVM/Logistic/RF/mixed linear model	Case–control study	C-index	MoCA	Recruit
20	Kang et al. (2022)	China	Diagnostic model	104		72	Random sampling 10-fold cross validation	32	10-fold cross validation	Logistic/SVM	Case–control study	C-index, Sensitivity and specificity	MoCA	Single-center
21	Shibata et al. (2022)	Japan	Diagnostic model	163	26	120	10-fold cross validation	43	cross validation	RF/XGBoost/lightGBM	Cohort study	C-index, Sensitivity and specificity	MoCA	multicenter
22	Chung et al. (2021)	Taiwan	Diagnostic model	116	81	87	4-fold cross validation	29	4-fold cross validation	ANN	Case–control study	C-index	MoCA or MMSE	Single-center
23	Chang et al. (2022)	China	Diagnostic model	57	116	–	–	–	–	logistic	Cohort study	C-index, Sensitivity and specificity	MoCA or MMSE	Single-center
24	Novak et al. (2021)	USA	Diagnostic model	20	34	20	–	–	–	logistic	Case–control study	C-index, Sensitivity and specificity	MoCA	Recruit
25	Gschwandtner et al. (2023)	Switzerland	Prediction model	75	14	–	–	–	–	CPM	Case–control study	C-index	MMSE	Recruit
26	Cai et al. (2021)	China	Diagnostic model	68	32	–	–	–	–	logistic	Case–control study	C-index, Sensitivity and specificity	MoCA	Recruit
27	Hou et al. (2024)	China	Prediction model	550	319	385	Bootstrap	165	cross validation	logistic	Case–control study	C-index, Sensitivity and specificity	MoCA	Single-center
28	Park et al. (2023)	Korea	Prediction model	262	75	168	External validation	94	10-fold cross validation	Cox	Cohort study	C-index, Sensitivity and specificity	MMSE	Multicenter
29	Sivaranjini and Sujatha (2024)	India	Diagnostic model	135	44	121	10-fold cross validation	14	10-fold cross validation	LR	Case–control study	--	MoCA	Single-center
30	Huang et al. (2024)	China	Prediction model	90	33	62	5-fold cross validation	18	5-fold cross validation	logistic	Cohort study	C-index, Sensitivity and specificity	MoCA	Multicenter
31	Baek et al. (2024)	Korea	Diagnostic model	1,293	591	1,202	–	91	5-fold cross validation	logistic	Case–control study	C-index, Sensitivity and specificity	MoCA	Multicenter
32	Parajuli et al. (2023a, 2023b)	USA	Diagnostic model	36	16	27	4-fold cross validation	9	4-fold cross validation	KNN	Case–control study	--	MoCA	Multicenter
33	Chen et al. (2023)	China	Diagnostic model	40	13	–	–	–	–	LR	Case–control study	C-index	MoCA	Single-center
34	McFall et al. (2023)	Canada	Prediction model	48	14	32	3-fold cross validation	16	3-fold cross validation	RF	Cohort study	C-index	DSM-V	multicenter
35	Zhang et al. (2023)	China	Prediction model	387	195	–	–	–	–	–	Cohort study	C-index, Sensitivity and specificity	DSM-V	multicenter
36	Gorji and Fathi Jouzdani (2024)	Iran	Prediction model	330	–	264	External validation	66	5-fold cross validation	RF	Cohort study	C-index, Sensitivity and specificity	MoCA	Single-center
37	Fiorenzato et al. (2024)	Italy	Diagnostic model	118	66	94	5-fold cross validation	24	5-fold cross validation	KNN/SVM/RF/XGBoost	Case–control study	–	MoCA or MMSE	Multicenter
38	Zhu et al. (2024)	China	Prediction model	141	72	99	5-fold cross validation	42	5-fold cross validation	RF	Cohort study	C-index, Sensitivity and specificity	MoCA or MMSE	Single-center
39	Beheshti et al. (2024)	Canada	Prediction model	104	–	78	10-fold cross validation	26	10-fold cross validation	SVM	Cohort study	Sensitivity and specificity	MoCA	Multicenter
40	Parajuli et al. (2023)	USA	Diagnostic model	36	16	27	4-fold cross validation	9	4-fold cross validation	KNN	Case–control study	C-index, Sensitivity and specificity	MoCA	Multicenter
41	Hosseinzadeh et al. (2023)	Canada	Prediction model	297	–	238	5-fold cross validation	59	5-fold cross validation	XGBoost	Cohort study	--	MoCA	Multicenter
42	Jian et al. (2024)	China	Prediction model	222	68	129	External validation	93	–	XGBoost	Cohort study	C-index, Sensitivity and specificity	MoCA	Multicenter
43	Li H. et al. (2025)	China	Prediction model	59	25	–	–	–	–	XGBoost	Case–control study	--	MMSE	Single-center
44	Luo et al. (2025)	China	Prediction model	99	48	69	5-fold cross validation	30	5-fold cross validation	RF/SVM	Cohort study	C-index, Sensitivity and specificity	MoCA	Multicenter
45	Liu et al. (2025)	China	Diagnostic model	60	30	54	10-fold cross validation	6	10-fold cross validation	LASSO	Case–control study	C-index, Sensitivity and specificity	MMSE	Single-center
46	d'Angremont et al. (2025)	Netherlands	Prediction model	34	–	31	leave-one-out cross-validation	4	leave-one-out cross-validation	Logistic	Case–control study	--	MoCA	Single-center
47	Li L. et al. (2025)	China	Prediction model	351	163	–	–	–	–	Cox	Case–control study	C-index, Sensitivity and specificity	MoCA	Recruit
48	Silva-Rodríguez et al. (2025)	Spain	Diagnostic model	85	32	57	Bootstrap	28	3-fold cross validation	Logistic	Case–control study	C-index, Sensitivity and specificity	MoCA	Recruit
49	Beheshti et al. (2024)	Canada	Prediction model	244	52	139	10-fold cross validation	105	10-fold cross validation	XGBoost	Cohort study	--	MoCA	Multicenter
50	Putha et al. (2025)	USA	Prediction model	174	–	122	10-fold cross validation	52	10-fold cross validation	Cox/LASSO	Cohort study	C-index, Sensitivity and specificity	MoCA	Multicenter
51	Kemp et al. (2025)	USA	Diagnostic model	33	15	26	10-fold cross validation	7	10-fold cross validation	XGBoost	Case–control study	C-index, Sensitivity and specificity	MoCA	Multicenter
52	Mostile et al. (2025)	Italy	Prediction model	79	26	71	10-fold cross validation	8	10-fold cross validation	KNN/RF	Cohort study	–	DSM-V	Multicenter

### 3.3 Risk of bias in studies

**Figure 2:**
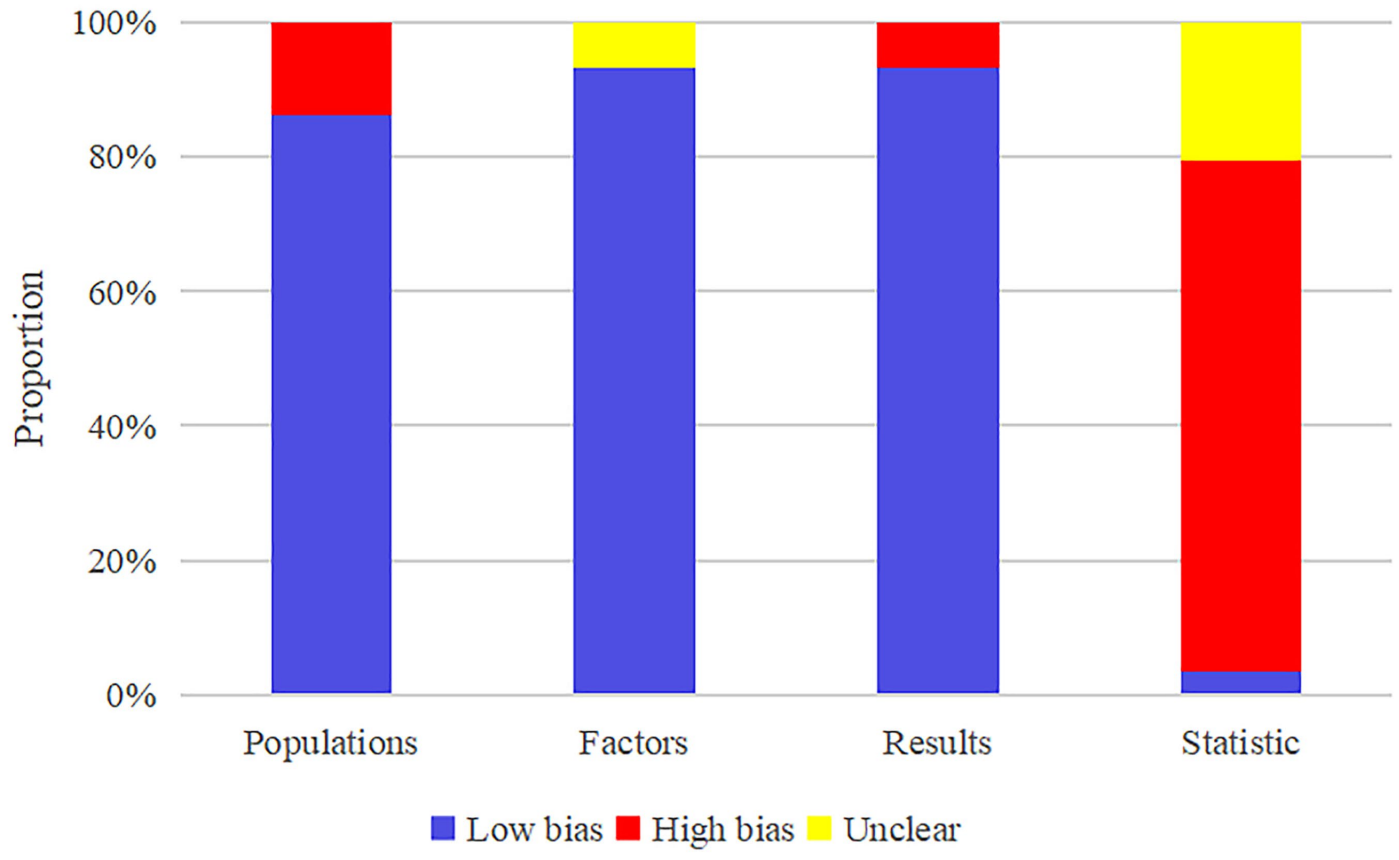
Results of risk of bias assessment of the included models.

All studies implemented feature selection and dimensionality reduction to mitigate overfitting. In terms of model evaluation, most reported discriminative statistics such as ROC curves, c-indices, and areas under the curve (AUCs), along with statistically significant measures (e.g., *p*-values, confidence intervals [CI]), whereas calibration metrics were less frequently provided. Multivariable analyses of clinical data were conducted in eight studies (Hogue et al., 2018; Zhang et al., 2020; Ortelli et al., 2022; Baek et al., 2024; McFall et al., 2023; d'Angremont et al., 2025; Beheshti et al., 2024; Putha et al., 2025) to develop MLMs, while another eight (Chen et al., 2022; Shen et al., 2022; Chung et al., 2021; Hou et al., 2024; Zhang et al., 2023; Li L. et al., 2025; Silva-Rodríguez et al., 2025; Mostile et al., 2025) incorporated both clinical characteristics and biological markers. Two studies (Parajuli et al., 2023a; Liu et al., 2023) incorporated clinical features alongside electrooculography and electromyography, and five (Koch et al., 2019; Novak et al., 2021; Cai et al., 2021; Fiorenzato et al., 2024; Parajuli et al., 2023b) combined clinical features with EEG signals. Twelve studies (Chen et al., 2023; Abós et al., 2017; Shibata et al., 2022; Gschwandtner et al., 2023; Sivaranjini and Sujatha, 2024; Huang et al., 2024; Gorji and Fathi Jouzdani, 2024; Beheshti and Ko, 2024; Hosseinzadeh et al., 2023; Jian et al., 2024; Li H. et al., 2025; Kemp et al., 2025) utilized both clinical and imaging features, three (Zhang et al., 2022; Brien et al., 2023; Chang et al., 2022) combined clinical data with ocular characteristics, and two (Russo et al., 2023; Amboni et al., 2022) integrated clinical data with gait parameters. Two studies (Tang et al., 2021; Park et al., 2023) developed MLMs utilizing clinical data and radiomic features, one (Liu et al., 2017) built an MLM incorporating clinical data and genetic scores, while another (García et al., 2021) utilized clinical data and speech domain features. Integrated MLMs were developed by combining imaging with EEG signals in one study (Zhang et al., 2021), and by merging clinical variables with acoustic parameters in another (Liu et al., 2025). Two studies (Schrag et al., 2017; Zhu et al., 2024) constructed MLMs employing imaging and biomarkers. A comprehensive MLM was created in one study (Kang et al., 2022) by integrating clinical data, radiomics, and combined radiomic and imaging features. Another two studies (Harvey et al., 2022; Luo et al., 2025) developed integrated MLMs via clinical data, biomarkers, and genetic scores. One study (Chen et al., 2021) presented a comprehensive MLM that combined clinical features, imaging, biomarkers, and genetic scores. Additionally, two studies (Zhang et al., 2022; Abós et al., 2017) performed cutoff value analyses to assess the risk related to the diagnostic and predictive accuracy of MLMs. The potential clinical utility of these MLMs was evaluated through decision curve analysis in three studies (Zhang et al., 2022; Chen et al., 2021; Kang et al., 2022). Nevertheless, no cost-effectiveness analyses were reported. The absence of a well-established gold standard for clinically diagnosing PD-CI presents a challenge in evaluating the concordance between developed MLMs and current gold standard approaches. Regarding open science and data sharing, the majority of studies did not make their source code publicly available (Figure 2).

### 3.4 Meta-analysis

#### 3.4.1 Diagnostic models

**Figure 3:**
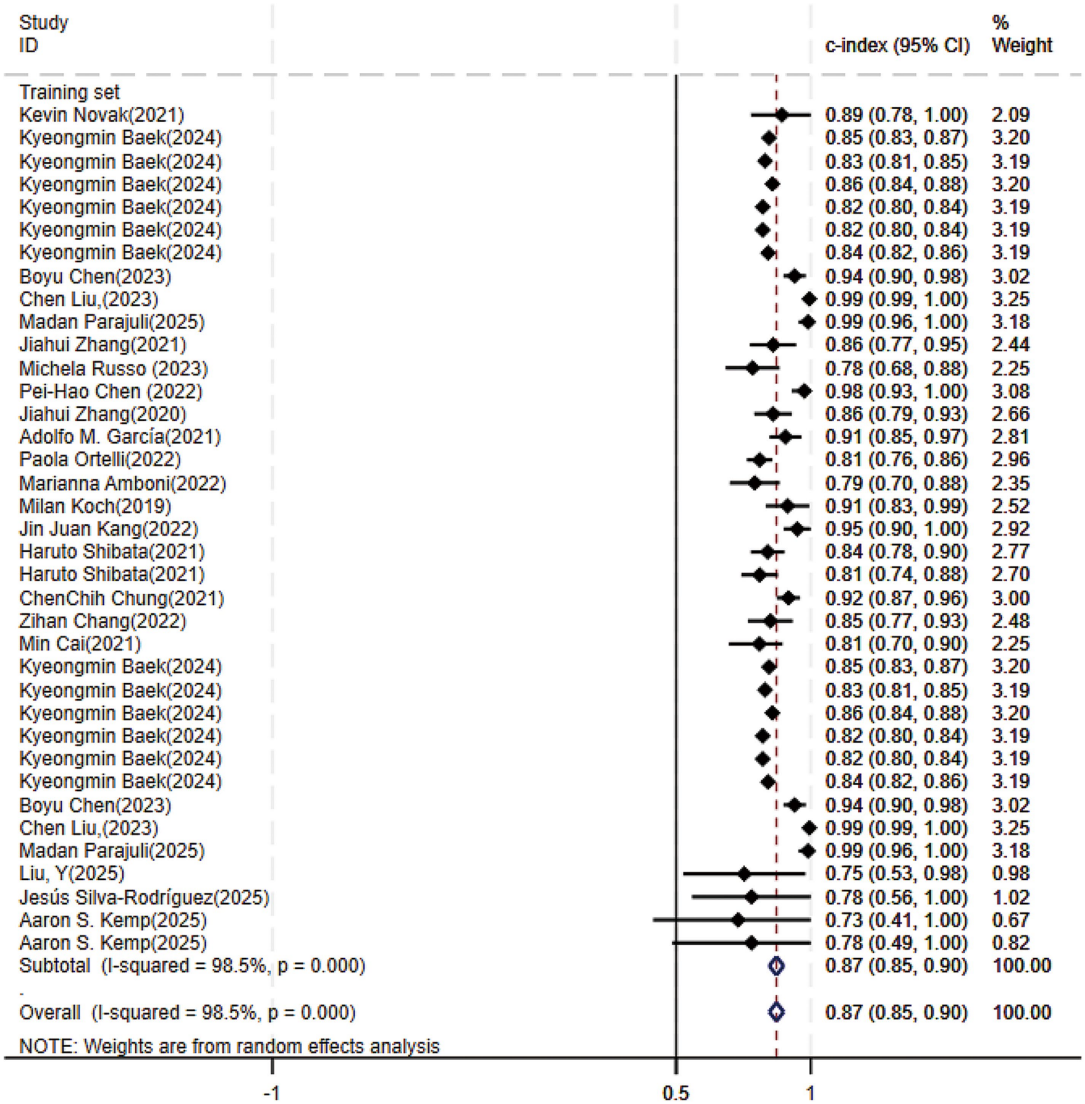
Forest plot of the meta-analysis of c-index for machine learning in the diagnosis of PD-CI in the training set.

**Figure 4:**
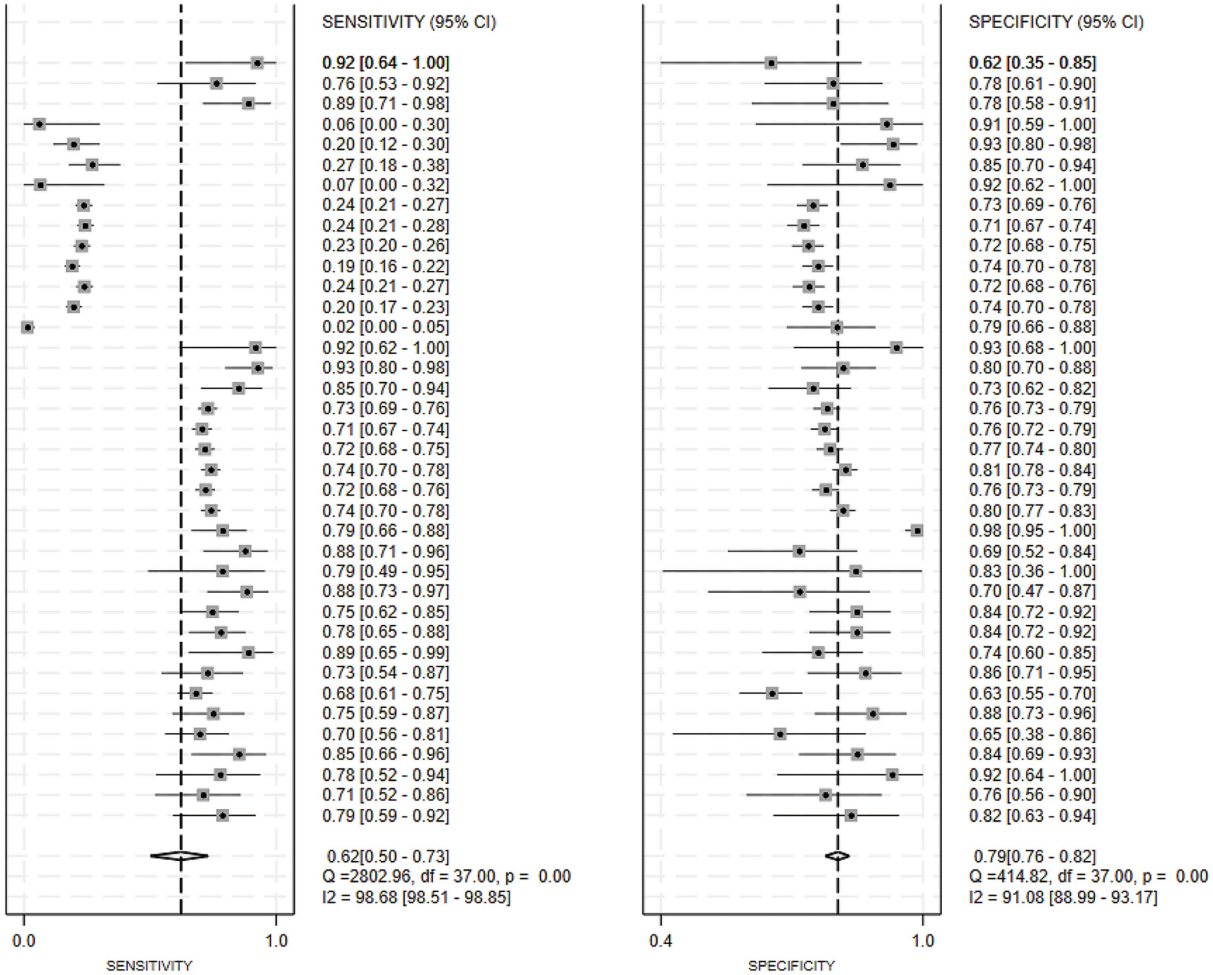
Forest plot of the meta-analysis of sensitivity and specificity for machine learning in the diagnosis of PD-CI in the training set.

**Figure 5:**
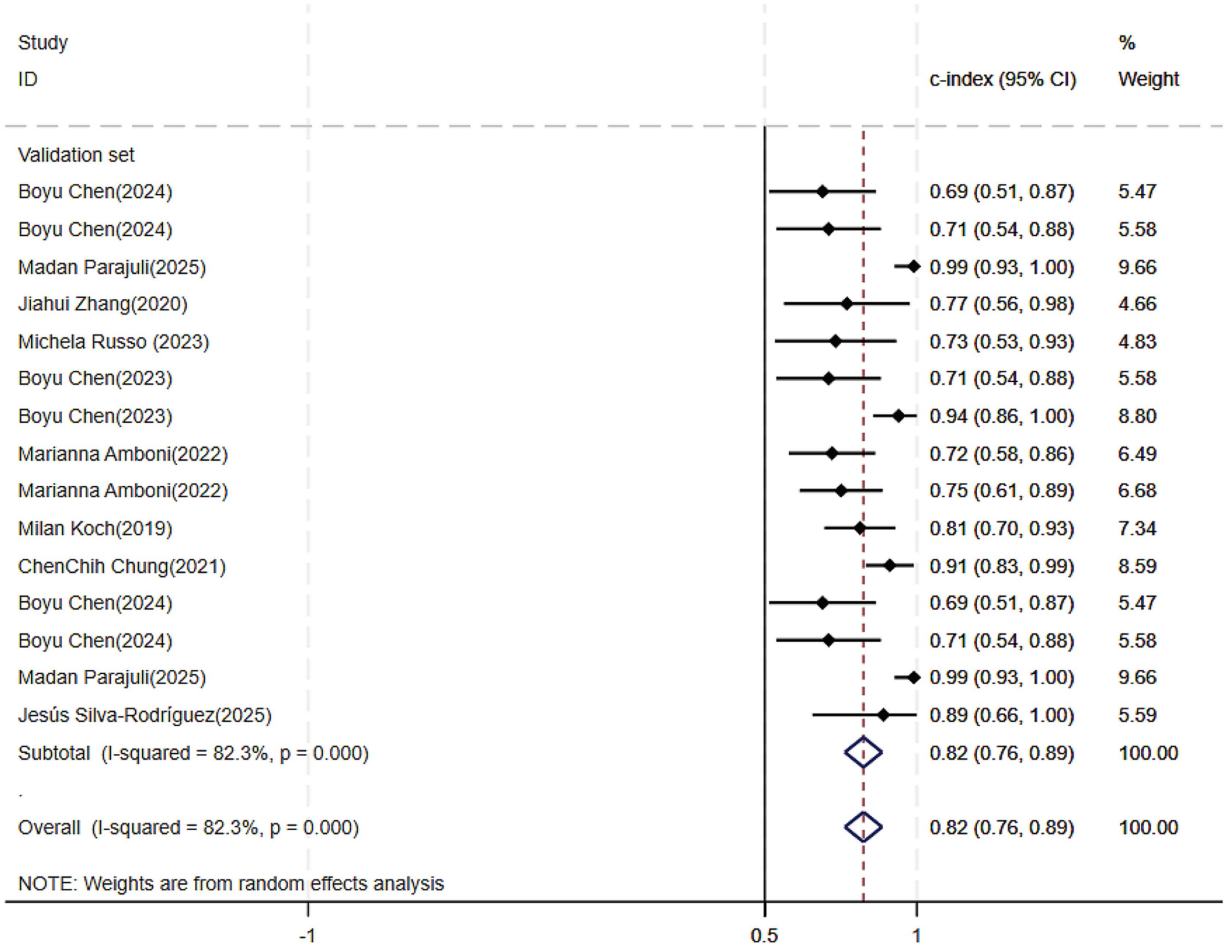
Forest plot of the meta-analysis of c-index for machine learning in the diagnosis of PD-CI in the validation set.

**Figure 6:**
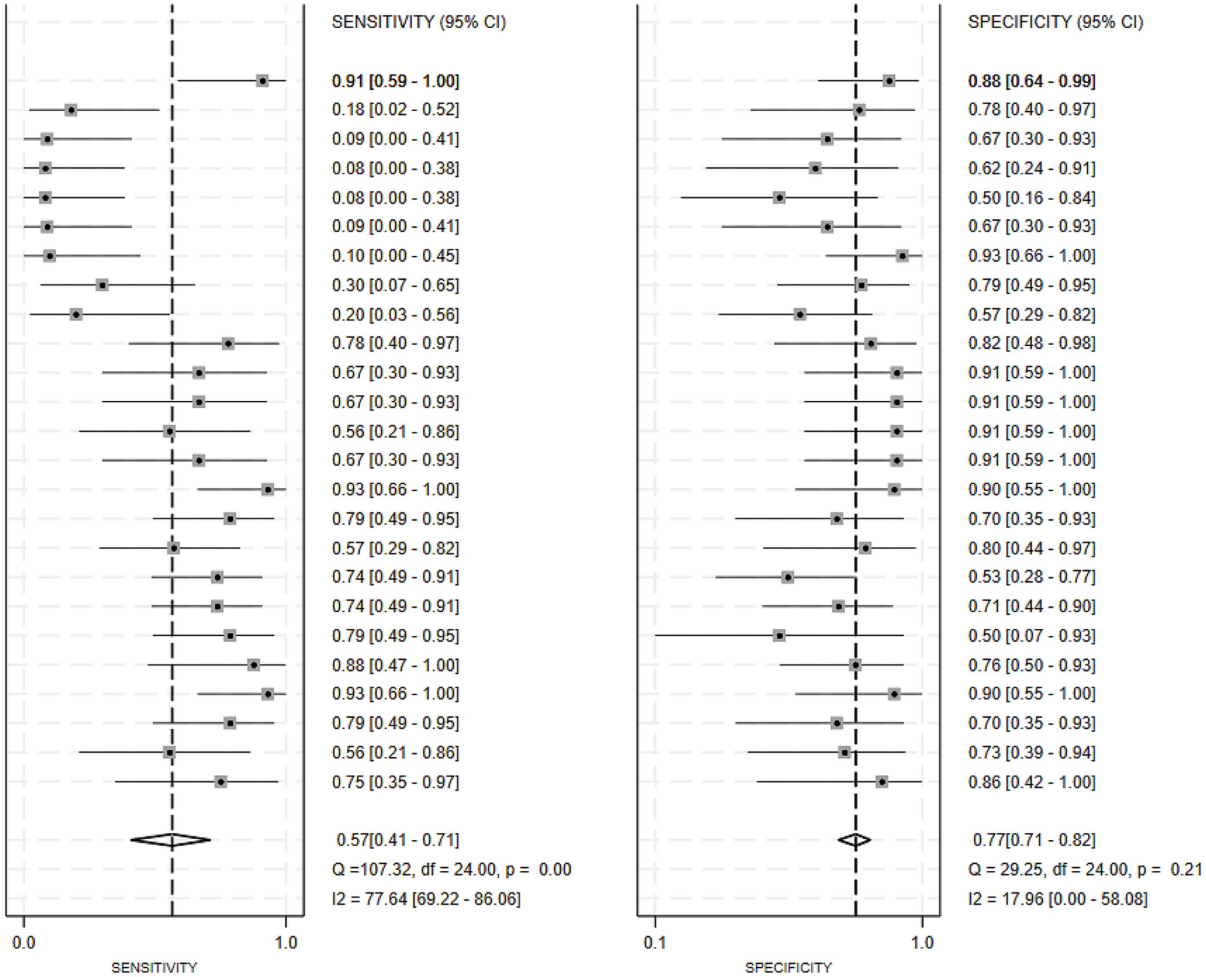
Forest plot of the meta-analysis of sensitivity and specificity for machine learning in the diagnosis of PD-CI in the validation set.

Of the 25 studies on PD-CI diagnostic models, 22 reported c-indices, and 19 reported c-indices, sensitivity, and specificity. A total of 66 models were included. A meta-analysis of the training datasets revealed 39 models with a mean c-index of 0.87 (95% CI: 0.85–0.90), a sensitivity of 0.62 (0.50–0.73), and a specificity of 0.79 (0.76–0.82) (Figures 3, 4). On validation datasets, 27 models reported these metrics as 0.82 (0.76–0.89), 0.57 (0.41–0.71), and 0.77 (0.71–0.82), respectively (Figures 5, 6).

Of all the constructed MLMs, the SVM and LR models demonstrated favorable diagnostic performance in the large-scale validation and training cohorts. Other models that warrant attention included K-Nearest Neighbors (KNN), XGBoost, RF, and the Least Absolute Shrinkage and Selection Operator (LASSO) model. Despite incorporating a limited number of models, the current study showed strong diagnostic accuracy. Incorporating a broader range of models in future research may help confirm their discriminatory precision.

#### 3.4.2 Prediction models

**Figure 7:**
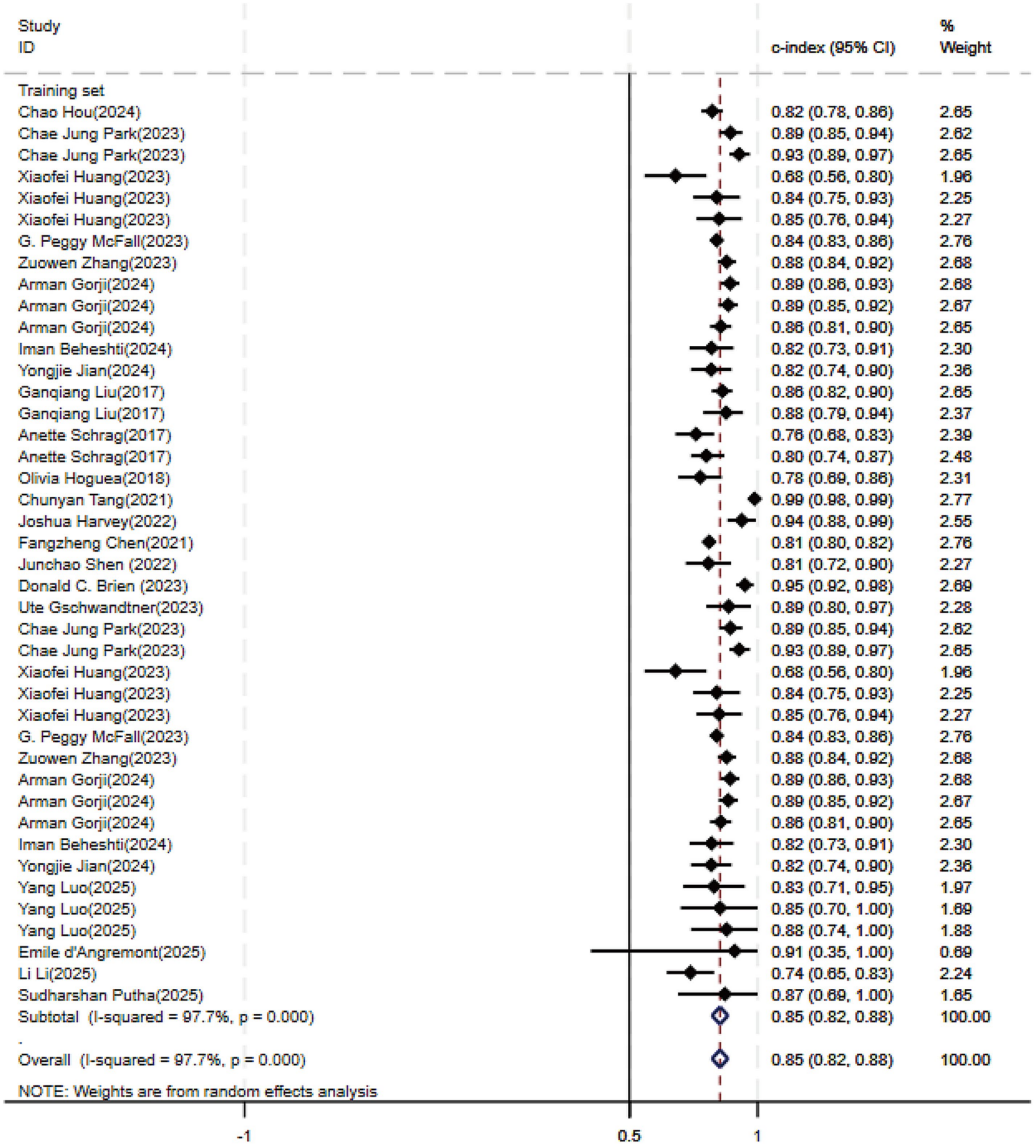
Forest plot of the meta-analysis of c-index for machine learning in the prediction of PD-CI in the training set.

**Figure 8:**
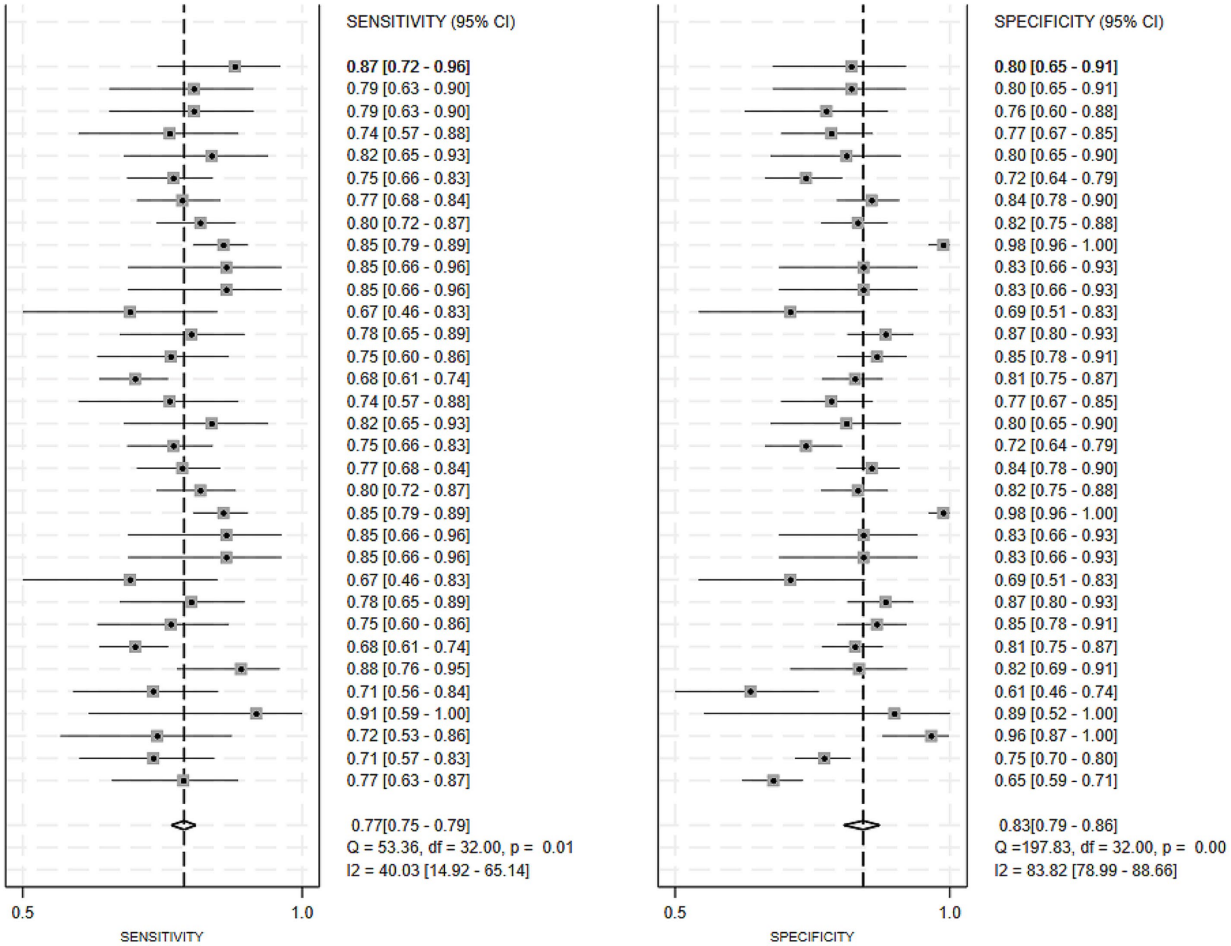
Forest plot of the meta-analysis of sensitivity and specificity for machine learning in the prediction of PD-CI in the training set.

**Figure 9:**
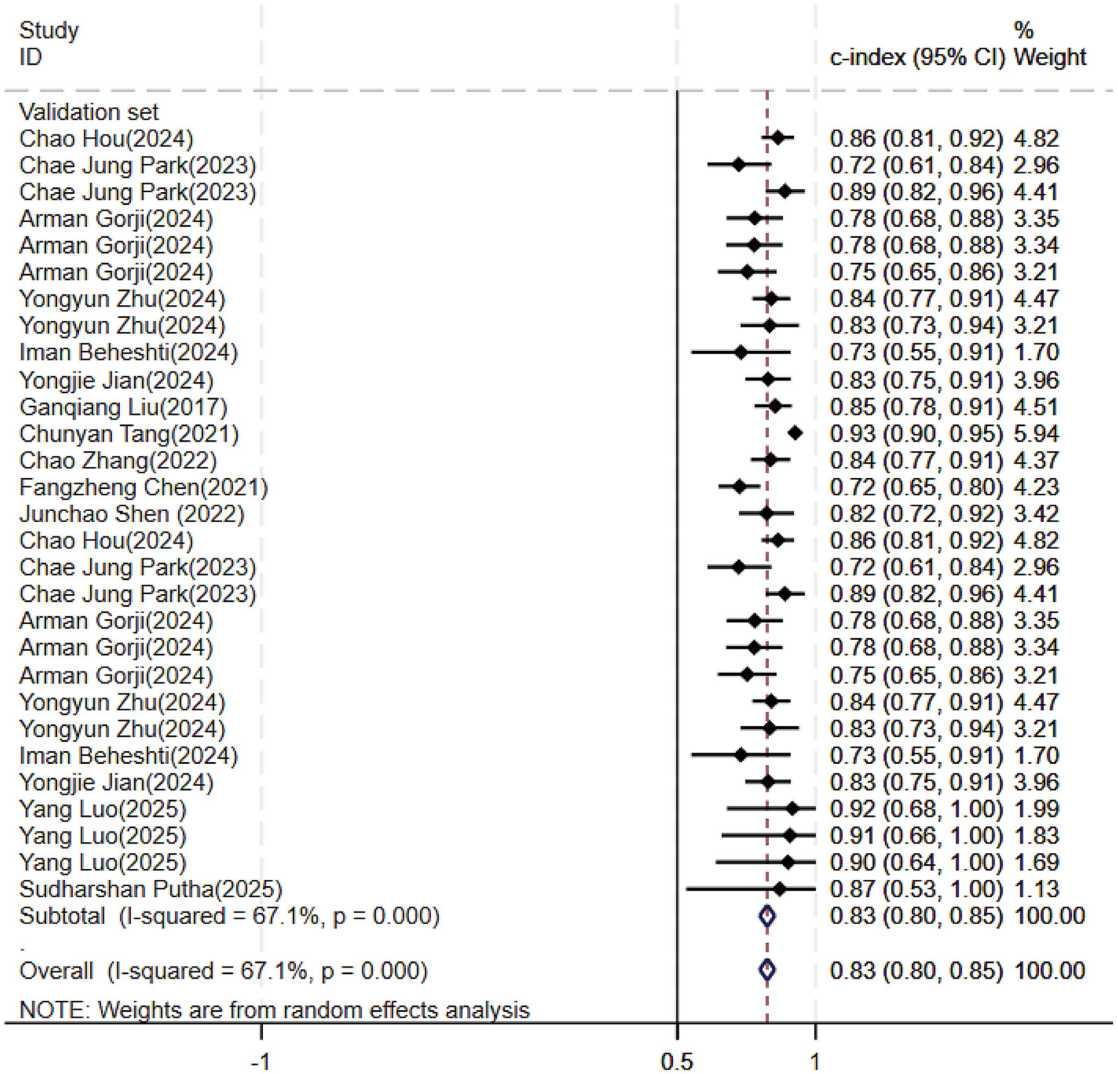
Forest plot of the meta-analysis of c-index for machine learning in the prediction of PD-CI in the validation set.

**Figure 10:**
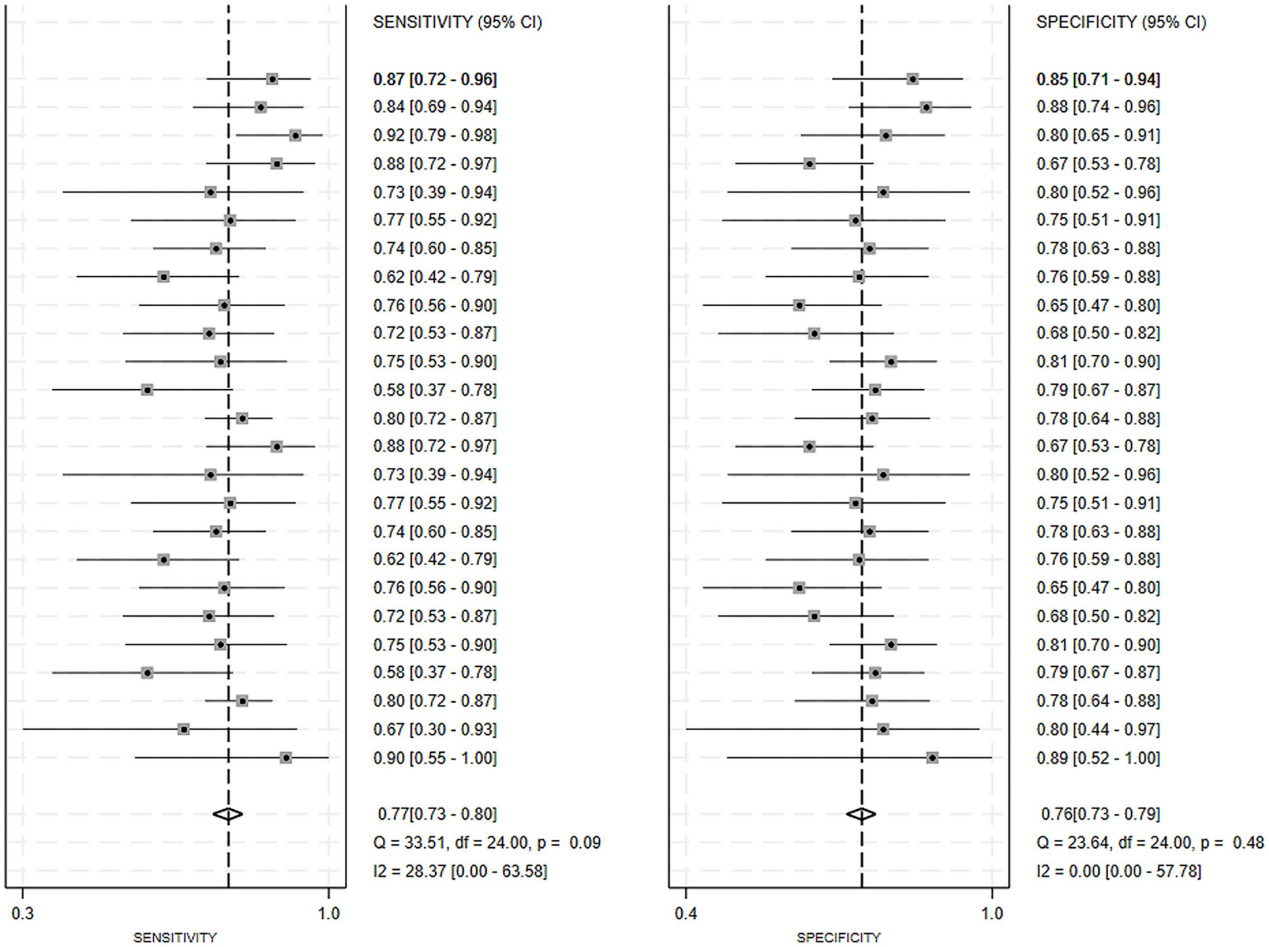
Forest plot of the meta-analysis of sensitivity and specificity for machine learning in the prediction of PD-CI in the validation set.

Regarding the 27 earlier studies that predicted PD-CI, 21 reported c-indices, 10 reported c-indices, sensitivity, and specificity, and one reported only sensitivity and specificity. A total of 72 models were included. The overall c-index for these models was 0.84, ranging from 0.81 to 0.87. In the training set, 43 MLMs reported a c-index, sensitivity, and specificity of 0.85 (0.82–0.88), 0.77 (0.75–0.79), and 0.83 (0.79–0.86) (Figures 7, 8), respectively. In the validation set, 29 MLMs illustrated a c-index of 0.83 (0.80–0.85), with sensitivity of 0.77 (0.73–0.80) and specificity of 0.76 (0.73–0.79). Cox regression and LR models displayed robust predictive performance in both the validation and training sets. Other models, including RF and LASSO, also warranted attention. Although the number of models incorporated was small, the current study still demonstrated good predictive accuracy (Figures 9–10).

#### 3.4.3 Sensitivity analysis and publication bias

Publication bias was evaluated quantitatively via funnel plots and Egger’s test to assess model stability. The results indicated no significant bias in the training set for diagnostic models (Egger’s test: *p* = 0.489), but revealed bias in the validation set (Egger’s test: *p* = 0.000) (Supplementary materials 3–6). For predictive models, significant publication bias was noted in both sets (Egger’s test: *p* = 0.000 for both) (Supplementary materials 7–10). The trim-and-fill method was employed to adjust for publication bias in the validation set of the diagnostic models and the training set of the predictive models, respectively, as shown in Supplementary materials 11, 12.

The reliability of the predictive and diagnostic models for PD-CI was examined through sensitivity analysis. The findings indicated that omitting each individual study had no substantial impact on the overall predictive and diagnostic outcomes for PD-CI, as shown in Supplementary materials 13–16.

Furthermore, no evidence of overfitting was detected in any of the diagnostic or predictive MLMs for PD-CI. Despite the relatively small number of models included in this study, they exhibited commendable predictive and diagnostic accuracy.

#### 3.4.4 Visualization of yearly publication volume and ML modeling variables

**Figure 11:**
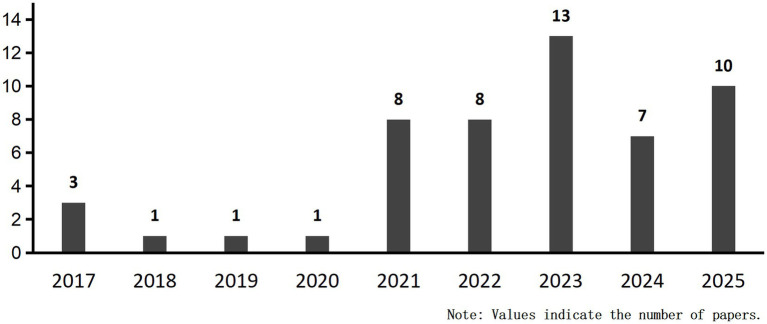
Visualize the number of papers published in this study each year.

**Figure 12:**
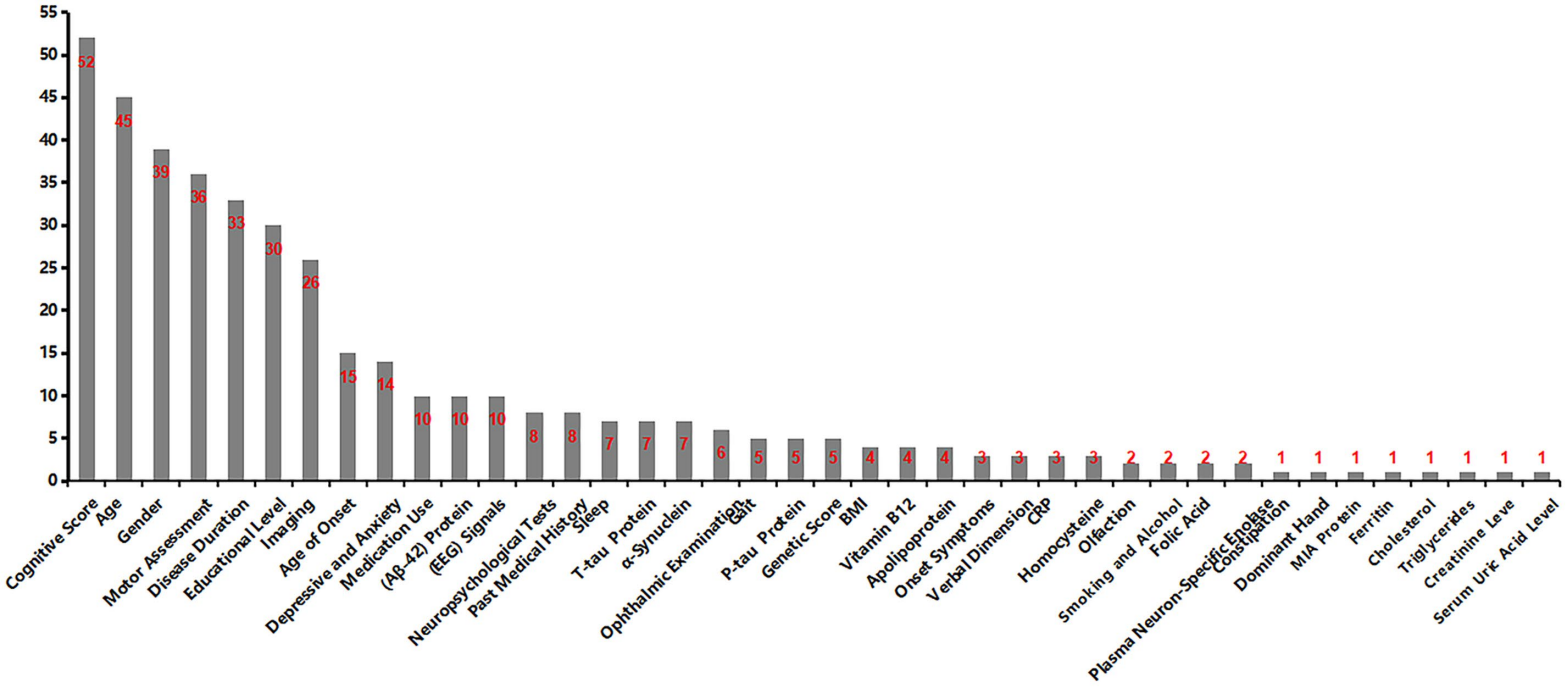
Visualization of variables for machine learning modeling.

Our systematic review of 52 publications revealed an upward trend in publication volume overall (Figure 11). By August 2025, ten articles had been included, reflecting the accelerating integration of ML into PD cognitive prediction and diagnosis. ML integrates high-dimensional data such as imaging, genetics, and behavior, employing complementary algorithms to automatically mine deep features for precise diagnosis and early warning. Concurrently, a comprehensive compilation of diagnostic and predictive markers for PD-CI was performed (Figure 12). The findings indicated that current research primarily relies on clinical and imaging data, while emerging biomarkers and digital assessment methods are gaining attention.

## 4 Discussion

### 4.1 Performance of ML in PD-CI assessment

The results of our meta-analysis indicated that the overall c-index of the diagnostic models was 0.85. The c-index, sensitivity, and specificity were 0.87, 0.62, and 0.79, respectively, in the training set, and 0.82, 0.57, and 0.77, respectively, in the validation set. ML techniques demonstrated commendable accuracy in the diagnosis of PD-CI. For the predictive model, the overall c-index stood at 0.84, with a c-index for the validation and training sets being 0.83 and 0.85, respectively. Overall, ML approaches exhibited strong predictive performance for PD-CI.

### 4.2 Advantages of other detection methods for PD-CI

Recently, significant progress has been achieved in the prediction and diagnosis of PD-CI. Concurrently, imaging techniques, along with auxiliary modalities such as taste, gait, and eye characteristics, have seen rapid development (Bian et al., 2023; Sheng et al., 2021). A review by Sun et al. (2023) revealed that imaging techniques utilizing surface-based morphometry (SBM) detected thinning in the frontal and temporal cortex. Notably, cortical thinning in the frontostriatal region serves as a high-risk biomarker for detecting a persistent cognitive decline in patients with cognitive impairment, offering robust predictive and diagnostic capabilities for PD-CI. A review by Oppo et al. (2020) has demonstrated that taste disturbances in PD patients mirror cortical involvement, co-occur with mild cognitive impairment, and can serve as a predictive and supplementary diagnostic tool for PD-CI. Monaghan et al. (2023) systematic review highlighted that PD patients with freezing of gait exhibited diminished cognitive abilities compared to those without freezing of gait across domains like overall cognition, executive function/attention, language, memory, and visual space, underscoring the significance of gait in predicting and diagnosing PD-CI. Tao et al. (2020)systematic review suggested that eye tracking tasks, particularly saccade tasks, can be employed to gather oculomotor nerve data during cognitive assessments. As an adjunct to traditional cognitive assessment scales, this method holds promise for PD-CI prediction and supplementary diagnosis. The findings from these studies suggest that both predictive and diagnostic models for PD-CI based on various variables exhibit commendable performance. However, comprehensive research on MLMs for PD-CI prediction remains limited, with scant discussion on holistic models leveraging AI. Despite significant advancements in the prediction and diagnosis methods for PD-CI, integrating them with artificial intelligence can enhance efficiency, lower detection costs, and further improve diagnostic and predictive accuracy in this condition.

### 4.3 Findings of this study

This research aims to systematically evaluate and synthesize evidence on the use of ML for initial detection and forecasting of PD-CI through a meta-analytic approach. Prior research has demonstrated that EEG signals (Novak et al., 2021; Gschwandtner et al., 2023), imaging (DAT imaging), genetic scores (Liu et al., 2017), and ocular characteristics can serve as significant predictors (Zhang et al., 2022; Brien et al., 2023) in the construction of multivariate models to improve the accuracy of disease prediction. The accuracy of models ranges from 80 to 84% (Zhang et al., 2021; Amboni et al., 2022; Koch et al., 2019). The integrated SVM model, based on the susceptibility values (MSV) and radiomics features of the substantia nigra and striatum system, demonstrated an accuracy rate of 95%. Furthermore, its polymorphic model exhibited superior diagnostic performance (Kang et al., 2022). The seven included studies (Liu et al., 2017; Tang et al., 2021; Zhang et al., 2022; Chen et al., 2021; Koch et al., 2019; Brien et al., 2023; Kang et al., 2022) utilized a combination of EEG, imaging, clinical features (such as demographic data, biomarkers, genetic scores, and speech and gait characteristics), and radiomics to construct comprehensive classification models. These models exhibited superior predictive and diagnostic performance. Additionally, it was demonstrated that the multimodal MLM outperformed the model based on a single biomarker (Wu et al., 2019). Consequently, future explorations of ML methodologies should integrate additional pertinent variables to develop more robust multimodal models. This could be incorporated into the multivariate features of existing prediction and diagnostic models to improve the precision of PD diagnosis and early identification. However, the interpretation of EEG and images predominantly depends on the expertise of clinical specialists. The process of detection is intricate and costly, thereby restricting its applicability, particularly in economically and medically underdeveloped regions. Consequently, developing a straightforward, non-invasive, and precise evaluation method based on MLMs holds great promise for predicting and diagnosing PD-CI. This approach could significantly enhance the prediction and diagnosis of the early risk of numerous diseases.

This study revealed that the dorsal-lateral prefrontal cortex (DLPFC) is a crucial region linked to cognitive impairment in PD. The abnormalities in the PMFG-type functional connectivity pattern and changes in the theta band can be assessed using EEG, offering a novel, significant biomarker for PD-CI. Furthermore, radiomics features situated in the temporal and frontal lobes, hippocampus, caudate nucleus, and thalamus—including morphological features and advanced features such as GLCM and GLRLM—exhibited strong detection and diagnostic performance. Additionally, CoMDA (Cognitive Assessment of Movement Disorders) applies AI in cognitive-screening assessments to generate a prediction of the cognitive profile; it is a useful and time-saving cognitive-screening tool that can accurately classify PD-CI.

The MLMs included in the present study integrated multimodal data, including demographics, serology, genetics, imaging, and electroencephalography, enabling objective screening of key biomarkers. This effectively overcomes the limitations of traditional clinical scales, such as strong subjectivity and limited dimensions, to achieve individualized risk prediction and auxiliary diagnosis for PD-MCI, providing precise data support for early intervention. A literature review revealed that clinical features such as age, disease duration, motor symptoms, and education level are the most widely used and established core bases for current PD-MCI assessment. Secondly, although there is substantial evidence for traditional fluid biomarkers (e.g., Aβ42) and neuroimaging, research on more disease-specific markers (e.g., *α*-syn and p-Tau) is insufficient. Their diagnostic value requires further exploration in future studies. Conversely, despite limited literature, genetic scores, electroencephalography, and digital assessments based on behavior, such as gait, speech, and eye movements, show significant potential as noninvasive, dynamic monitoring tools and represent promising frontiers. In summary, future PD-MCI research should focus on constructing multimodal, cross-scale, integrated predictive models. While consolidating the core position of clinical assessment, efforts must be made to validate specific biomarkers and promote the clinical translation of objective, convenient digital behavioral markers for the early and precise identification and dynamic monitoring of PD-MCI.

### 4.4 Strengths and limitations

Our research demonstrates that ML techniques exhibit high sensitivity and specificity in the early prediction and diagnosis of PD-CI. These findings can serve as evidence-based guidance for predicting, screening, and diagnosing cognitive impairment in PD, thereby significantly contributing to the disease’s predictive and clinical decision-making systems. Nevertheless, this meta-analysis has certain limitations. Firstly, the limited number of incorporated models and modeling variables restricts the analysis of the predictive and diagnostic performance of various models for PD-CI, thus impeding the comprehensive interpretation of our results. Secondly, some included studies were case–control studies, hindering accurate risk of bias assessment during variable evaluation. Due to these constraints, it is challenging to evaluate the significance of the number of detailed studies, modeling, and variables. A considerable portion of the original studies did not report the contribution rates of different modeling variables in the models, thereby limiting the thorough elaboration of results. Furthermore, there exists significant heterogeneity among the eligible studies. The adoption of various dimensionality reduction or feature selection techniques can introduce substantial heterogeneity across radiomic investigations. Further research is required to validate this observation. Moreover, due to the absence of standardized operating guidelines, ML methods continue to face numerous challenges in their current applications. Additionally, many relevant studies employ retrospective designs, and few studies have validation sets. Furthermore, most studies predominately employ internal validation or resampling techniques such as cross-validation, restricting the external applicability and clinical translation of MLMs. Consequently, it is imperative to gather imaging data from various hospitals and research sites in the future to ensure the external applicability of models. This will enable MLMs to be used in a broader spectrum of clinical scenarios.

### 4.5 Future direction

Despite the current advancements in ML-based approaches, they are not without inherent challenges and discrepancies during the actual model construction process. Future studies on ML are advised to construct multimodal models. However, the specific types of models and variables to be incorporated into these models necessitate further exploration. The integration of radiomics with imaging features enhances diagnostic accuracy, while the accuracy of predictions improves through the combination of biomarkers, genes, and clinical features. Nonetheless, these detection methods are not only inconvenient and costly but also heavily dependent on clinicians’ expertise and patients’ subjective judgment. Furthermore, we have noted pronounced correlations between certain extracted variables, complicating the selection of modeling variables. Consequently, it is crucial to use the most appropriate methods to select modeling variables, which can address the issue of overfitting. Further studies are desired to construct more refined ML methods by selecting more appropriate variables based on each model’s characteristics and advantages. This could facilitate more efficient, rapid, and accurate prediction and diagnosis of PD-CI.

## 5 Conclusion

Published ML research findings demonstrate that ML has exceptional accuracy and application potential in predicting and differentiating PD and PD-MCI. Predictive models primarily utilize interpretable clinical data and genetic features as core variables, while diagnostic models integrate clinical, neuroimaging, and radiomic data. By combining multimodal information from imaging, genetics, and behavior, MLMs exhibit stable, high discriminative performance in independent validation cohorts and significantly outperform traditional clinical scales. They can also identify individuals at high risk of converting to PD-MCI at an earlier stage. By leveraging noninvasive data acquisition methods, such as wearable devices and speech signals, combined with CNN architectures and self-supervised pre-training techniques, MLMs have surpassed the predictive efficacy of serum biomarkers. This reduces reliance on invasive or costly tests, such as lumbar punctures and PET imaging, and expands assessment dimensions while substantially lowering costs. Furthermore, feature importance analysis has identified a refined combination of “plasma protein biomarkers + polygenic risk score,” which might replace traditional cerebrospinal fluid detection methods and offer a scalable pathway for large-scale population screening. Notably, existing studies did not observe model overfitting, providing valuable insights for developing future risk prediction and intelligent diagnostic tools and further confirming the immense potential of ML in advancing PD and related disease diagnosis and prediction. However, limitations persist. Only a minority of the original studies included had independent validation datasets, which may have influenced the interpretation of the findings. Subsequent research must address this issue systematically to improve the robustness and clinical value of the conclusions.

## Data Availability

The original contributions presented in the study are included in the article/Supplementary material, further inquiries can be directed to the corresponding author.

## References

[ref1] Aarsland D. Andersen K. Larsen J. P. Lolk A. Kragh-Sørensen P. (2003). Prevalence and characteristics of dementia in Parkinson disease: an 8-year prospective study. Arch. Neurol. 60, 387–392. doi: 10.1001/archneur.60.3.387 12633150

[ref2] Abós A. Baggio H. C. Segura B. García-Díaz A. I. Compta Y. Martí M. J. . (2017). Discriminating cognitive status in Parkinson's disease through functional connectomics and machine learning. Sci. Rep. 7:45347. doi: 10.1038/srep45347, 28349948 PMC5368610

[ref3] Agosta F. Canu E. Stefanova E. Sarro L. Tomić A. Špica V. . (2014). Mild cognitive impairment in Parkinson's disease is associated with a distributed pattern of brain white matter damage. Hum. Brain Mapp. 35, 1921–1929. doi: 10.1002/hbm.22302, 23843285 PMC6869219

[ref4] Amboni M. Ricciardi C. Adamo S. Nicolai E. Volzone A. Erro R. . (2022). Machine learning can predict mild cognitive impairment in Parkinson's disease. Front. Neurol. 13:1010147. doi: 10.3389/fneur.2022.1010147, 36468069 PMC9714435

[ref5] Arumugam K. Naved M. Shinde P. P. Leiva-Chauca O. Huaman-Osorio A. Gonzales-Yanac T. (2023). Multiple disease prediction using machine learning algorithms. Mater Today Proc 80, 3682–3685. doi: 10.1016/j.matpr.2021.07.361, 41311396

[ref6] Baek K. Kim Y. M. Na H. K. Lee J. Shin D. H. Heo S. J. . (2024). Comparing Montreal cognitive assessment performance in Parkinson's disease patients: age- and education-adjusted cutoffs vs. machine learning. J. Mov. Disord. 17, 171–180. doi: 10.14802/jmd.23271, 38346940 PMC11082615

[ref7] Beheshti I. Ko J. H. (2024). Predicting the occurrence of mild cognitive impairment in Parkinson's disease using structural MRI data. Front. Neurosci. 18:1375395. doi: 10.3389/fnins.2024.1375395, 38699676 PMC11063344

[ref8] Beheshti I. Perron J. Ko J. (2024). Euroanatomical signature of the transition from normal cognition to MCI in Parkinson's disease. Aging Dis. 16, 619–632. doi: 10.14336/AD.2024.0323 38913040 PMC11745458

[ref9] Berg D. Postuma R. B. Adler C. H. Bloem B. R. Chan P. Dubois B. . (2015). MDS research criteria for prodromal Parkinson's disease. Mov. Disord. 30, 1600–1611. doi: 10.1002/mds.26431, 26474317

[ref10] Bhatt C. M. Patel P. Ghetia T. Mazzeo P. L. (2023). Effective heart disease prediction using machine learning techniques. Algorithms 16:88. doi: 10.3390/a16020088

[ref11] Bian J. Wang X. Hao W. Zhang G. Wang Y. (2023). The differential diagnosis value of radiomics-based machine learning in Parkinson's disease: a systematic review and meta-analysis. Front. Aging Neurosci. 15:1199826. doi: 10.3389/fnagi.2023.1199826, 37484694 PMC10357514

[ref12] Brandão P. R. P. Munhoz R. P. Grippe T. C. Cardoso F. E. C. de Almeida E. C. B. M. Titze-de-Almeida R. . (2020). Cognitive impairment in Parkinson's disease: a clinical and pathophysiological overview. J. Neurol. Sci. 419:117177. doi: 10.1016/j.jns.2020.117177, 33068906

[ref13] Brien D. C. Riek H. C. Yep R. Huang J. Coe B. Areshenkoff C. . (2023). Classification and staging of Parkinson's disease using video-based eye tracking. Parkinsonism Relat. Disord. 110:105316. doi: 10.1016/j.parkreldis.2023.105316, 36822878

[ref14] Cai M. Dang G. Su X. Zhu L. Shi X. Che S. . (2021). Identifying mild cognitive impairment in Parkinson's disease with electroencephalogram functional connectivity. Front. Aging Neurosci. 13:701499. doi: 10.3389/fnagi.2021.701499, 34276350 PMC8281812

[ref15] Chang Z. Xie F. Li H. Yuan F. Zeng L. Shi L. . (2022). Retinal nerve Fiber layer thickness and associations with cognitive impairment in Parkinson's disease. Front. Aging Neurosci. 14:832768. doi: 10.3389/fnagi.2022.832768, 35222000 PMC8867012

[ref16] Chen P. H. Hou T. Y. Cheng F. Y. Shaw J. S. (2022). Prediction of cognitive degeneration in Parkinson's disease patients using a machine learning method. Brain Sci. 12. doi: 10.3390/brainsci12081048, 36009111 PMC9405552

[ref17] Chen F. Li Y. Ye G. Zhou L. Bian X. Liu J. (2021). Development and validation of a prognostic model for cognitive impairment in Parkinson's disease with REM sleep behavior disorder. Front. Aging Neurosci. 13:703158. doi: 10.3389/fnagi.2021.703158, 34322014 PMC8311737

[ref18] Chen B. Xu M. Yu H. He J. Li Y. Song D. . (2023). Detection of mild cognitive impairment in Parkinson's disease using gradient boosting decision tree models based on multilevel DTI indices. J. Transl. Med. 21:310. doi: 10.1186/s12967-023-04158-8, 37158918 PMC10165759

[ref19] Chung C. C. Chan L. Chen J. H. Bamodu O. A. Chiu H. W. Hong C. T. (2021). Plasma extracellular vesicles tau and β-amyloid as biomarkers of cognitive dysfunction of Parkinson's disease. FASEB J. 35:e21895. doi: 10.1096/fj.202100787R, 34478572

[ref20] d'Angremont E. Renken R. van der Zee S. de Vries E. F. J. van Laar T. Sommer I. E. C. (2025). Cholinergic denervation patterns in Parkinson's disease associated with cognitive impairment across domains. Hum. Brain Mapp. 46:e70047. doi: 10.1002/hbm.70047, 39846322 PMC11755113

[ref21] Debray T. P. Damen J. A. Riley R. D. Snell K. Reitsma J. B. Hooft L. . (2019). A framework for meta-analysis of prediction model studies with binary and time-to-event outcomes. Stat. Methods Med. Res. 28, 2768–2786. doi: 10.1177/0962280218785504, 30032705 PMC6728752

[ref22] Delgado-Alvarado M. Gago B. Navalpotro-Gomez I. Jiménez-Urbieta H. Rodriguez-Oroz M. C. (2016). Biomarkers for dementia and mild cognitive impairment in Parkinson's disease. Mov. Disord. 31, 861–881. doi: 10.1002/mds.26662, 27193487

[ref23] Fiorenzato E. Moaveninejad S. Weis L. Biundo R. Antonini A. Porcaro C. (2024). Brain dynamics complexity as a signature of cognitive decline in Parkinson's disease. Mov. Disord. 39, 305–317. doi: 10.1002/mds.29678, 38054573

[ref24] García A. M. Arias-Vergara T. Cv C. Nöth E. Schuster M. Welch A. E. . (2021). Cognitive determinants of dysarthria in Parkinson's disease: an automated machine learning approach. Mov. Disord. 36, 2862–2873. doi: 10.1002/mds.28751 34390508

[ref25] GBD 2016 Neurology Collaborators (2019). Global, regional, and national burden of neurological disorders, 1990-2016: a systematic analysis for the global burden of disease study 2016. Lancet Neurol. 18, 459–480. doi: 10.1016/S1474-4422(18)30499-X, 30879893 PMC6459001

[ref26] Goldman J. G. Sieg E. (2020). Cognitive impairment and dementia in Parkinson disease. Clin. Geriatr. Med. 36, 365–377. doi: 10.1016/j.cger.2020.01.001, 32222308

[ref27] Gorji A. Fathi Jouzdani A. (2024). Machine learning for predicting cognitive decline within five years in Parkinson's disease: comparing cognitive assessment scales with DAT SPECT and clinical biomarkers. PLoS One 19:e0304355. doi: 10.1371/journal.pone.0304355 39018311 PMC11253925

[ref28] Gschwandtner U. Bogaarts G. Roth V. Fuhr P. (2023). Prediction of cognitive decline in Parkinson's disease (PD) patients with electroencephalography (EEG) connectivity characterized by time-between-phase-crossing (TBPC). Sci. Rep. 13:5093. doi: 10.1038/s41598-023-32345-6, 36991083 PMC10060251

[ref29] Harvey J. Reijnders R. A. Cavill R. Duits A. Köhler S. Eijssen L. . (2022). Machine learning-based prediction of cognitive outcomes in de novo Parkinson's disease. NPJ Parkinsons Dis. 8:150. doi: 10.1038/s41531-022-00409-5, 36344548 PMC9640625

[ref30] Hogue O. Fernandez H. H. Floden D. P. (2018). Predicting early cognitive decline in newly-diagnosed Parkinson's patients: a practical model. Parkinsonism Relat. Disord. 56, 70–75. doi: 10.1016/j.parkreldis.2018.06.031, 29936131 PMC6425482

[ref31] Hosseinzadeh M. Gorji A. Fathi Jouzdani A. Rezaeijo S. M. Rahmim A. Salmanpour M. R. (2023). Prediction of cognitive decline in Parkinson's disease using clinical and DAT SPECT imaging features, and hybrid machine learning systems. Diagnostics 13. doi: 10.3390/diagnostics13101691, 37238175 PMC10217464

[ref32] Hou C. Yang F. Li S. Ma H. Y. Li F. X. Zhang W. . (2024). A nomogram based on neuron-specific enolase and substantia nigra hyperechogenicity for identifying cognitive impairment in Parkinson's disease. Quant. Imaging Med. Surg. 14, 3581–3592. doi: 10.21037/qims-23-1778, 38720848 PMC11074765

[ref33] Huang X. He Q. Ruan X. Li Y. Kuang Z. Wang M. . (2024). Structural connectivity from DTI to predict mild cognitive impairment in de novo Parkinson's disease. Neuroimage Clin. 41:103548. doi: 10.1016/j.nicl.2023.103548, 38061176 PMC10755095

[ref34] Janvin C. C. Larsen J. P. Aarsland D. Hugdahl K. (2006). Subtypes of mild cognitive impairment in Parkinson's disease: progression to dementia. Mov. Disord. 21, 1343–1349. doi: 10.1002/mds.20974, 16721732

[ref35] Jian Y. Peng J. Wang W. Hu T. Wang J. Shi H. . (2024). Prediction of cognitive decline in Parkinson's disease based on MRI radiomics and clinical features: a multicenter study. CNS Neurosci. Ther. 30:e14789. doi: 10.1111/cns.14789, 38923776 PMC11196371

[ref36] Kang J. J. Chen Y. Xu G. D. Bao S. L. Wang J. Ge M. . (2022). Combining quantitative susceptibility mapping to radiomics in diagnosing Parkinson's disease and assessing cognitive impairment. Eur. Radiol. 32, 6992–7003. doi: 10.1007/s00330-022-08790-8, 35461376

[ref37] Kemp A. S. Eubank A. J. Younus Y. Galvin J. E. Prior F. W. Larson-Prior L. J. (2025). Sequential patterning of dynamic brain states distinguish Parkinson's disease patients with mild cognitive impairments. Neuroimage Clin. 46:103779. doi: 10.1016/j.nicl.2025.103779, 40252310 PMC12033993

[ref38] Koch M Geraedts V Wang H Tannemaat M Bäck T Automated machine learning for EEG-based classification of Parkinson's disease patients. 2019 IEEE International Conference on Big Data (Big Data) (2019) p. 4845–4852.

[ref39] Kubota K. J. Chen J. A. Little M. A. (2016). Machine learning for large-scale wearable sensor data in Parkinson's disease: concepts, promises, pitfalls, and futures. Mov. Disord. 31, 1314–1326. doi: 10.1002/mds.26693, 27501026

[ref40] Li H. Shao X. Jia J. Wang B. Wang J. Liu K. . (2025). A multi-modal study on cerebrovascular dysfunction in cognitive decline of de novo Parkinson's disease. Neuroimage Clin. 48:103836. doi: 10.1016/j.nicl.2025.103836, 40633451 PMC12275129

[ref41] Li L. Tang S. Hao B. Gao X. Liu H. Wang B. . (2025). Early detection and Management of Cognitive Impairment in Parkinson's disease: a predictive model approach. Brain Behav. 15:e70423. doi: 10.1002/brb3.70423, 40079619 PMC11904965

[ref42] Liu C. Jiang Z. Liu S. Chu C. Wang J. Liu W. . (2023). Frequency-dependent microstate characteristics for mild cognitive impairment in Parkinson's disease. IEEE Trans. Neural Syst. Rehabil. Eng. 31, 4115–4124. doi: 10.1109/TNSRE.2023.3324343, 37831557

[ref43] Liu G. Locascio J. J. Corvol J. C. Boot B. Liao Z. Page K. . (2017). Prediction of cognition in Parkinson's disease with a clinical-genetic score: a longitudinal analysis of nine cohorts. Lancet Neurol. 16, 620–629. doi: 10.1016/S1474-4422(17)30122-9, 28629879 PMC5761650

[ref44] Liu Y. Wang X. X. Wang X. J. Yin M. M. Tan M. Y. Wang C. P. . (2025). Acoustic prosodic parameters associated with Parkinson's disease cognitive impairment. Parkinsonism Relat. Disord. 132:107306. doi: 10.1016/j.parkreldis.2025.107306, 39893775

[ref45] Luo Y. Xiang Y. Liu J. Hu Y. Guo J. (2025). A multi-omics framework based on machine learning as a predictor of cognitive impairment progression in early Parkinson's disease. Neurol Ther. 14, 643–658. doi: 10.1007/s40120-025-00716-y, 39985630 PMC11906927

[ref46] McFall G. P. Bohn L. Gee M. Drouin S. M. Fah H. Han W. . (2023). Identifying key multi-modal predictors of incipient dementia in Parkinson's disease: a machine learning analysis and tree SHAP interpretation. Front. Aging Neurosci. 15:1124232. doi: 10.3389/fnagi.2023.1124232, 37455938 PMC10347530

[ref47] Moher D. Shamseer L. Clarke M. Ghersi D. Liberati A. Petticrew M. . (2015). Preferred reporting items for systematic review and meta-analysis protocols (PRISMA-P) 2015 statement. Syst. Rev. 4:1. doi: 10.1186/2046-4053-4-1, 25554246 PMC4320440

[ref48] Monaghan A. S. Gordon E. Graham L. Hughes E. Peterson D. S. Morris R. (2023). Cognition and freezing of gait in Parkinson's disease: a systematic review and meta-analysis. Neurosci. Biobehav. Rev. 147:105068. doi: 10.1016/j.neubiorev.2023.105068, 36738813

[ref49] Mostile G. Contrafatto F. Terranova R. Terravecchia C. Luca A. Sinitò M. . (2023). Turning and sitting in early parkinsonism: differences between idiopathic Normal pressure hydrocephalus associated with parkinsonism and Parkinson's disease. Mov. Disord. Clin. Pract. 10, 466–471. doi: 10.1002/mdc3.13638, 36949785 PMC10026280

[ref50] Mostile G. Quattropani S. Contrafatto F. Terravecchia C. Caci M. R. Chiara A. . (2025). Testing machine learning algorithms to evaluate fluctuating and cognitive profiles in Parkinson's disease by motion sensors and EEG data. Comput. Struct. Biotechnol. J. 27, 778–784. doi: 10.1016/j.csbj.2025.02.019, 40092665 PMC11910500

[ref51] Mukherjee A. Biswas A. Roy A. Biswas S. Gangopadhyay G. Das S. K. (2017). Behavioural and psychological symptoms of dementia: correlates and impact on caregiver distress. Dement. Geriatr. Cogn. Disord. Extra 7, 354–365. doi: 10.1159/000481568, 29282408 PMC5731149

[ref52] Nemade D. Subramanian T. Shivkumar V. (2021). An update on medical and surgical treatments of Parkinson's disease. Aging Dis. 12, 1021–1035. doi: 10.14336/AD.2020.1225, 34221546 PMC8219497

[ref53] Novak K. Chase B. A. Narayanan J. Indic P. Markopoulou K. (2021). Quantitative electroencephalography as a biomarker for cognitive dysfunction in Parkinson's disease. Front. Aging Neurosci. 13:804991. doi: 10.3389/fnagi.2021.804991, 35046794 PMC8761986

[ref54] Oppo V. Melis M. Melis M. Tomassini Barbarossa I. Cossu G. (2020). "smelling and tasting" Parkinson's disease: using senses to improve the knowledge of the disease. Front. Aging Neurosci. 12:43. doi: 10.3389/fnagi.2020.00043, 32161534 PMC7052524

[ref55] Ortelli P. Ferrazzoli D. Versace V. Cian V. Zarucchi M. Gusmeroli A. . (2022). Optimization of cognitive assessment in Parkinsonisms by applying artificial intelligence to a comprehensive screening test. NPJ Parkinsons Dis. 8:42. doi: 10.1038/s41531-022-00304-z, 35410449 PMC9001753

[ref56] Parajuli M. Amara A. Shaban M. (2023a). Deep-learning detection of mild cognitive impairment from sleep electroencephalography for patients with Parkinson's disease. PLoS One 18:e0286506. doi: 10.1371/journal.pone.0286506, 37535549 PMC10399849

[ref57] Parajuli M Amara AW Shaban M. Screening of mild cognitive impairment in patients with Parkinson's disease using a Variational mode decomposition based deep-learning. 2023 11th international IEEE/EMBS conference on neural engineering (NER), Baltimore, MD, USA. (2023) 1–6. doi: 10.1109/NER52421.2023.10123759

[ref58] Park C. J. Eom J. Park K. S. Park Y. W. Chung S. J. Kim Y. J. . (2023). An interpretable multiparametric radiomics model of basal ganglia to predict dementia conversion in Parkinson's disease. NPJ Parkinsons Dis. 9:127. doi: 10.1038/s41531-023-00566-1, 37648733 PMC10468504

[ref59] Pringsheim T. Jette N. Frolkis A. Steeves T. D. (2014). The prevalence of Parkinson's disease: a systematic review and meta-analysis. Mov. Disord. 29, 1583–1590. doi: 10.1002/mds.25945, 24976103

[ref60] Putha S. Gayam S. R. Kasaraneni B. P. Kondapaka K. K. Nallamala S. K. Thuniki P. (2025). Neuroscience-informed nomogram model for early prediction of cognitive impairment in Parkinson's disease. Neurosci. Inform. 5:100189. doi: 10.1016/j.neuri.2025.100189

[ref61] Reitsma J. B. Glas A. S. Rutjes A. W. Scholten R. J. Bossuyt P. M. Zwinderman A. H. (2005). Bivariate analysis of sensitivity and specificity produces informative summary measures in diagnostic reviews. J. Clin. Epidemiol. 58, 982–990. doi: 10.1016/j.jclinepi.2005.02.022, 16168343

[ref62] Russo M. Amboni M. Barone P. Pellecchia M. T. Romano M. Ricciardi C. . (2023). Identification of a gait pattern for detecting mild cognitive impairment in Parkinson's disease. Sensors 23. doi: 10.3390/s23041985, 36850582 PMC9963713

[ref63] Schrag A. Siddiqui U. F. Anastasiou Z. Weintraub D. Schott J. M. (2017). Clinical variables and biomarkers in prediction of cognitive impairment in patients with newly diagnosed Parkinson's disease: a cohort study. Lancet Neurol. 16, 66–75. doi: 10.1016/S1474-4422(16)30328-3, 27866858 PMC5377592

[ref64] Shen J. Amari N. Zack R. Skrinak R. T. Unger T. L. Posavi M. . (2022). Plasma MIA, CRP, and albumin predict cognitive decline in Parkinson's disease. Ann. Neurol. 92, 255–269. doi: 10.1002/ana.26410, 35593028 PMC9329215

[ref65] Sheng L. Zhao P. Ma H. Radua J. Yi Z. Shi Y. . (2021). Cortical thickness in Parkinson's disease: a coordinate-based meta-analysis. Aging (Albany NY) 13, 4007–4023. doi: 10.18632/aging.202368, 33461168 PMC7906199

[ref66] Shibata H. Uchida Y. Inui S. Kan H. Sakurai K. Oishi N. . (2022). Machine learning trained with quantitative susceptibility mapping to detect mild cognitive impairment in Parkinson's disease. Parkinsonism Relat. Disord. 94, 104–110. doi: 10.1016/j.parkreldis.2021.12.004, 34906915

[ref67] Silva-Rodríguez J. Labrador-Espinosa M. Castro-Labrador S. Muñoz-Delgado L. Franco-Rosado P. Castellano-Guerrero A. M. . (2025). Imaging biomarkers of cortical neurodegeneration underlying cognitive impairment in Parkinson's disease. Eur. J. Nucl. Med. Mol. Imaging 52, 2002–2014. doi: 10.1007/s00259-025-07070-z, 39888421 PMC12014801

[ref68] Sivaranjini S. Sujatha C. M. (2024). Analysis of cognitive dysfunction in Parkinson's disease using voxel based morphometry and radiomics. Cogn. Process. 25, 521–532. doi: 10.1007/s10339-024-01197-x, 38714621

[ref69] Sun W. Shi X. Fan Y. Wang C. Wang X. Wang Y. . (2023). Research progress of MRI in cognitive impairment of Parkinson's disease. Chin. J. Magn. Reson. Imaging. 14, 134–138. doi: 10.12015/issn.1674-8034.2023.07.024

[ref70] Tang C. Zhao X. Wu W. Zhong W. Wu X. (2021). An individualized prediction of time to cognitive impairment in Parkinson's disease: a combined multi-predictor study. Neurosci. Lett. 762:136149. doi: 10.1016/j.neulet.2021.136149, 34352339

[ref71] Tao L. Wang Q. Liu D. Wang J. Zhu Z. Feng L. (2020). Eye tracking metrics to screen and assess cognitive impairment in patients with neurological disorders. Neurol. Sci. 41, 1697–1704. doi: 10.1007/s10072-020-04310-y, 32125540

[ref72] Valasaki M. (2023). Constructing the detecting stage: social processes and the diagnostic journey of early onset Parkinson's disease. Sociol. Health Illn. 45, 872–889. doi: 10.1111/1467-9566.13622, 36762824

[ref73] Wu Y. Jiang J. H. Chen L. Lu J. Y. Ge J. J. Liu F. T. . (2019). Use of radiomic features and support vector machine to distinguish Parkinson's disease cases from normal controls. Ann Transl. Med. 7:773. doi: 10.21037/atm.2019.11.26, 32042789 PMC6990013

[ref74] Zhang J. Gao Y. He X. Feng S. Hu J. Zhang Q. . (2021). Identifying Parkinson's disease with mild cognitive impairment by using combined MR imaging and electroencephalogram. Eur. Radiol. 31, 7386–7394. doi: 10.1007/s00330-020-07575-1, 33389038

[ref75] Zhang J. Li Y. Gao Y. Hu J. Huang B. Rong S. . (2020). An SBM-based machine learning model for identifying mild cognitive impairment in patients with Parkinson's disease. J. Neurol. Sci. 418:117077. doi: 10.1016/j.jns.2020.117077, 32798842

[ref76] Zhang Z. Li S. Wang S. (2023). Application of periventricular white matter Hyperintensities combined with homocysteine into predicting mild cognitive impairment in Parkinson's disease. Int. J. Gen. Med. 16, 785–792. doi: 10.2147/IJGM.S399307, 36879618 PMC9985451

[ref77] Zhang C. Wu Q. Q. Hou Y. Wang Q. Zhang G. J. Zhao W. B. . (2022). Ophthalmologic problems correlates with cognitive impairment in patients with Parkinson's disease. Front. Neurosci. 16:928980. doi: 10.3389/fnins.2022.928980, 36278010 PMC9583907

[ref78] Zhu Y. Wang F. Ning P. Zhu Y. Zhang L. Li K. . (2024). Multimodal neuroimaging-based prediction of Parkinson's disease with mild cognitive impairment using machine learning technique. NPJ Parkinsons Dis. 10:218. doi: 10.1038/s41531-024-00828-6, 39528560 PMC11555067

