# Supplementary material for: Machine learning methods for the detection and prediction of cognitive impairment in Parkinson’s disease: a systematic review and meta-analysis
Source: Front Aging Neurosci. 2025 Dec 11;17:1704039. doi: 10.3389/fnagi.2025.1704039 (PMC12738896; doi:10.3389/fnagi.2025.1704039)
Supplement: Supplementary file 1 [file Supplementary_file_1.docx]

**Supplementary materials**

**Supplementary Material 1.** PRISMA checklist.

**Supplementary Material 2.** Literature search strategy.

**Supplementary Material 3.** Funnel plot of the diagnostic models in the training set

**Supplementary Material 4.** Egger’s test for the diagnostic models in the training set

**Supplementary Material 5.** Funnel plot of the diagnostic models in the validation set

**Supplementary Material 6.** Egger’s test for the diagnostic models in the validation set

**Supplementary Material 7.** Funnel plot of the prediction models in the training set

**Supplementary Material 8.** Egger’s test for the prediction models in the training set

**Supplementary Material 9.** Funnel plot of the prediction models in the validation set

**Supplementary Material 10.** Egger’s test for the prediction models in the validation set

**Supplementary Material 11.** Trim-and-fill method for the diagnostic models in the validation set

**Supplementary Material 12.** Trim-and-fill method for the prediction models in the training set

**Supplementary Material 13.** Sensitivity analysis of the diagnostic models in the training set

**Supplementary Material 14.** Sensitivity analysis of the diagnostic models in the validation set

**Supplementary Material 15.** Sensitivity analysis of the prediction models in the training set

**Supplementary Material 16.** Sensitivity analysis of the prediction models in the validation set

**Supplementary Material 1.** PRISMA checklist.

| **Section and Topic** | **Item #** | **Checklist item** | **Location where item is reported** |
| --- | --- | --- | --- |
| **TITLE** | | |  |
| Title | 1 | Identify the report as a systematic review | Title |
| **ABSTRACT** | | |  |
| Abstract | 2 | See the PRISMA 2020 for Abstracts checklist. | Abstract |
| **INTRODUCTION** | | |  |
| Rationale | 3 | Describe the rationale for the review in the context of existing knowledge. | Introduction |
| Objectives | 4 | Provide an explicit statement of the objective(s) or question(s) the review addresses. | Introduction |
| **METHODS** | | |  |
| Eligibility criteria | 5 | Specify the inclusion and exclusion criteria for the review and how studies were grouped for the syntheses. | Methods- Eligibility criteria |
| Information sources | 6 | Specify all databases, registers, websites, organisations, reference lists and other sources searched or consulted to identify studies. Specify the date when each source was last searched or consulted. | Methods-Information sources |
| Search strategy | 7 | Present the full search strategies for all databases, registers and websites, including any filters and limits used. | Methods-Search strategy |
| Selection process | 8 | Specify the methods used to decide whether a study met the inclusion criteria of the review, including how many reviewers screened each record and each report retrieved, whether they worked independently, and if applicable, details of automation tools used in the process. | Methods-Selection process |
| Data collection process | 9 | Specify the methods used to collect data from reports, including how many reviewers collected data from each report, whether they worked independently, any processes for obtaining or confirming data from study investigators, and if applicable, details of automation tools used in the process. | Methods-Data collection process |
| Data items | 10a | List and define all outcomes for which data were sought. Specify whether all results that were compatible with each outcome domain in each study were sought (e.g. for all measures, time points, analyses), and if not, the methods used to decide which results to collect. | Methods-Data items |
|  | 10b | List and define all other variables for which data were sought (e.g. participant and intervention characteristics, funding sources). Describe any assumptions made about any missing or unclear information. | Methods-Data items |
| Study risk of bias assessment | 11 | Specify the methods used to assess risk of bias in the included studies, including details of the tool(s) used, how many reviewers assessed each study and whether they worked independently, and if applicable, details of automation tools used in the process. | Methods-Study risk of bias assessment |
| Effect measures | 12 | Specify for each outcome the effect measure(s) (e.g. risk ratio, mean difference) used in the synthesis or presentation of results. | Methods-Effect measures |
| Synthesis methods | 13a | Describe the processes used to decide which studies were eligible for each synthesis (e.g. tabulating the study intervention characteristics and comparing against the planned groups for each synthesis (item #5)). | Methods-Synthesis methods |
|  | 13b | Describe any methods required to prepare the data for presentation or synthesis, such as handling of missing summary statistics, or data conversions. | Methods-Synthesis methods |
|  | 13c | Describe any methods used to tabulate or visually display results of individual studies and syntheses. | Methods-Synthesis methods |
|  | 13d | Describe any methods used to synthesize results and provide a rationale for the choice(s). If meta-analysis was performed, describe the model(s), method(s) to identify the presence and extent of statistical heterogeneity, and software package(s) used. | Methods-Synthesis methods |
|  | 13e | Describe any methods used to explore possible causes of heterogeneity among study results (e.g. subgroup analysis, meta-regression). | Methods-Synthesis methods |
|  | 13f | Describe any sensitivity analyses conducted to assess robustness of the synthesized results. | Methods-Synthesis methods |
| Reporting bias assessment | 14 | Describe any methods used to assess risk of bias due to missing results in a synthesis (arising from reporting biases). | Methods-Reporting bias assessment |
| Certainty assessment | 15 | Describe any methods used to assess certainty (or confidence) in the body of evidence for an outcome. | Methods-Certainty assessment |
| **RESULTS** | | |  |
| Study selection | 16a | Describe the results of the search and selection process, from the number of records identified in the search to the number of studies included in the review, ideally using a flow diagram. | Results-study selection |
|  | 16b | Cite studies that might appear to meet the inclusion criteria, but which were excluded, and explain why they were excluded. | Results-Study selection |
| Study characteristics | 17 | Cite each included study and present its characteristics. | Results-Study characteristics |
| Risk of bias in studies | 18 | Present assessments of risk of bias for each included study. | Results-Risk of bias in studies |
| Results of individual studies | 19 | For all outcomes, present, for each study: (a) summary statistics for each group (where appropriate) and (b) an effect estimate and its precision (e.g. confidence/credible interval), ideally using structured tables or plots. | Results-Results of individual studies |
| Results of syntheses | 20a | For each synthesis, briefly summarise the characteristics and risk of bias among contributing studies. | Results-Results of syntheses |
|  | 20b | Present results of all statistical syntheses conducted. If meta-analysis was done, present for each the summary estimate and its precision (e.g. confidence/credible interval) and measures of statistical heterogeneity. If comparing groups, describe the direction of the effect. | Results-Results of syntheses |
|  | 20c | Present results of all investigations of possible causes of heterogeneity among study results. | Results-Results of syntheses |
|  | 20d | Present results of all sensitivity analyses conducted to assess the robustness of the synthesized results. | Results-Results of syntheses |
| Reporting biases | 21 | Present assessments of risk of bias due to missing results (arising from reporting biases) for each synthesis assessed. | Results-Reporting biases |
| Certainty of evidence | 22 | Present assessments of certainty (or confidence) in the body of evidence for each outcome assessed. | Results-Certainty of evidence |
| **DISCUSSION** | | |  |
| Discussion | 23a | Provide a general interpretation of the results in the context of other evidence. | Discussion |
|  | 23b | Discuss any limitations of the evidence included in the review. | Discussion |
|  | 23c | Discuss any limitations of the review processes used. | Discussion |
|  | 23d | Discuss implications of the results for practice, policy, and future research. | Discussion |
| **OTHER INFORMATION** | | |  |
| Registration and protocol | 24a | Provide registration information for the review, including register name and registration number, or state that the review was not registered. | Methods-Study registration |
|  | 24b | Indicate where the review protocol can be accessed, or state that a protocol was not prepared. | Methods-Study registration |
|  | 24c | Describe and explain any amendments to information provided at registration or in the protocol. | Methods-Study registration |
| Support | 25 | Describe sources of financial or non-financial support for the review, and the role of the funders or sponsors in the review. | Funding |
| Competing interests | 26 | Declare any competing interests of review authors. | Acknowledgements |
| Availability of data, code and other materials | 27 | Report which of the following are publicly available and where they can be found: template data collection forms; data extracted from included studies; data used for all analyses; analytic code; any other materials used in the review. | Figure legends， Supplementary Materials and Table 1 |

**Supplementary Material 2.** Literature search strategy.

**1.Pubmed**

| Search number | Query | Results |
| --- | --- | --- |
| #1 | "Parkinson Disease"[MeSH Terms] | 91290 |
| #2 | "parkinson disease"[Title/Abstract] OR "parkinson s disease"[Title/Abstract] OR "primary parkinsonism"[Title/Abstract] OR "paralysis agitans"[Title/Abstract] OR "parkinsons disease"[Title/Abstract] OR "idiopathic parkinsonism"[Title/Abstract] OR "paralysis agitans"[Title/Abstract] OR "parkinson dementia complex"[Title/Abstract] | 136249 |
| #3 | #1 OR #2 | 149909 |
| #4 | "Machine Learning"[MeSH Terms] | 21116 |
| #5 | "machine learning"[Title/Abstract] OR "transfer learning"[Title/Abstract] OR "deep learning"[Title/Abstract] OR "hierarchical learning"[Title/Abstract] OR "ensemble learning"[Title/Abstract] OR "artificial intelligence"[Title/Abstract] OR "prediction model"[Title/Abstract] OR "random forest"[Title/Abstract] OR "neural network"[Title/Abstract] OR "ANN"[Title/Abstract] OR "support vector machine"[Title/Abstract] OR "SVM"[Title/Abstract] OR "gradient boosting machine"[Title/Abstract] OR "GBM"[Title/Abstract] OR "Nomogram"[Title/Abstract] OR "XGboost"[Title/Abstract] OR "Adaboost"[Title/Abstract] OR "decision tree"[Title/Abstract] OR "k nearest neighbor"[Title/Abstract] OR "external validation"[Title/Abstract] OR "Risk-Prediction"[Title/Abstract] OR "Risk-Prediction"[Title/Abstract] OR "Radiomics"[Title/Abstract] OR "radiomic"[Title/Abstract] OR "radiogenomic"[Title/Abstract] OR "radiomics-based"[Title/Abstract] OR "Texture"[Title/Abstract] | 523948 |
| #6 | #4 OR #5 | 539792 |
| #7 | "Cognitive Dysfunction"[MeSH Terms] | 93760 |
| #8 | "cognitive dysfunction"[Title/Abstract] OR "cognitive dysfunctions"[Title/Abstract] OR "cognitive impairments"[Title/Abstract] OR "cognitive impairment"[Title/Abstract] OR "cognitive disorder"[Title/Abstract] OR "cognitive disorders"[Title/Abstract] OR "mild cognitive impairment"[Title/Abstract] OR "mild cognitive impairments"[Title/Abstract] OR "cognitive decline"[Title/Abstract] OR "cognitive declines"[Title/Abstract] OR "mental deterioration"[Title/Abstract] OR "mental deteriorations"[Title/Abstract] OR "cognitive defect"[Title/Abstract] OR "cognitive defects"[Title/Abstract] OR "cognitive deficit"[Title/Abstract] OR "cognitive disability"[Title/Abstract] OR "cognitive dysfunction"[Title/Abstract] OR "overinclusion"[Title/Abstract] | 166613 |
| #9 | #7 OR #8 | 191053 |
| #10 | #3 AND #6 AND #9 | 272 |

**2.Cochrane**

| Search number | Query | Results |
| --- | --- | --- |
| #1 | MeSH descriptor: [Parkinson Disease] explode all trees | 6338 |
| #2 | (Parkinson Disease):ti,ab,kw OR (Parkinson's Disease):ti,ab,kw OR (Primary Parkinsonism):ti,ab,kw OR (Paralysis Agitans):ti,ab,kw OR (Parkinsons disease):ti,ab,kw | 12740 |
| #3 | (idiopathic parkinsonism):ti,ab,kw OR (paralysis agitans):ti,ab,kw OR (Parkinson dementia complex):ti,ab,kw | 1178 |
| #4 | #1 or #2 or #3 | 29330 |
| #5 | MeSH descriptor: [Machine Learning] explode all trees | 938 |
| #6 | (machine learning):ti,ab,kw OR (Transfer Learning):ti,ab,kw OR (Deep learning):ti,ab,kw OR (Hierarchical Learning):ti,ab,kw OR (Ensemble Learning):ti,ab,kw | 5612 |
| #7 | (Support vector machine):ti,ab,kw OR (SVM):ti,ab,kw OR (Gradient Boosting Machine):ti,ab,kw OR (GBM):ti,ab,kw OR (Nomogram):ti,ab,kw | 3342 |
| #8 | (XGboost):ti,ab,kw OR (Adaboost):ti,ab,kw OR (Decision tree):ti,ab,kw OR (K-Nearest Neighbor):ti,ab,kw OR (External validation):ti,ab,kw | 2582 |
| #9 | (Risk Prediction):ti,ab,kw OR (Risk-Prediction):ti,ab,kw OR (Radiomics):ti,ab,kw OR (radiomic):ti,ab,kw OR (radiogenomic):ti,ab,kw | 7096 |
| #10 | (radiomics-based):ti,ab,kw OR (Texture):ti,ab,kw | 2104 |
| #11 | (artificial intelligence):ti,ab,kw OR (Prediction model):ti,ab,kw OR (random forest):ti,ab,kw OR (neural network):ti,ab,kw OR (ANN):ti,ab,kw | 12031 |
| #12 | #5 or #6 or #7 or #8 or #9 or #10 or #11 | 24878 |
| #13 | MeSH descriptor: [Machine Learning] explode all trees | 938 |
| #14 | (Cognitive Dysfunction):ti,ab,kw OR (Cognitive Dysfunctions):ti,ab,kw OR (Cognitive Impairments):ti,ab,kw OR (Cognitive Impairment):ti,ab,kw OR (Cognitive Disorder):ti,ab,kw | 41093 |
| #15 | (Cognitive Disorders):ti,ab,kw OR (Mild Cognitive Impairment):ti,ab,kw OR (Mild Cognitive Impairments):ti,ab,kw OR (Cognitive Decline):ti,ab,kw OR (Cognitive Declines):ti,ab,kw | 32103 |
| #16 | (Mental Deterioration):ti,ab,kw OR (Mental Deteriorations):ti,ab,kw OR (cognitive defect):ti,ab,kw OR (cognitive defects):ti,ab,kw OR (cognitive deficit):ti,ab,kw | 9622 |
| #17 | (cognitive disability):ti,ab,kw OR (cognitive dysfunction):ti,ab,kw OR (overinclusion):ti,ab,kw OR (response interference):ti,ab,kw | 14559 |
| #18 | #13 or #14 or #15 or #16 or #17 | 58821 |
| #19 | #4 and #12 and #18 | 79 |

**3.Embase**

| Search number | Query | Results |
| --- | --- | --- |
| #1 | 'parkinson disease'/exp | 204243 |
| #2 | 'parkinson disease'/exp OR 'parkinson disease' OR (parkinson AND ('disease'/exp OR disease)) OR 'primary parkinsonism':ab,ti OR 'parkinsons disease':ab,ti OR 'idiopathic parkinsonism':ab,ti OR 'paralysis agitans':ab,ti OR 'parkinson dementia complex':ab,ti | 270758 |
| #3 | 'machine learning'/exp | 442331 |
| #4 | 'machine learning'/exp OR 'machine learning' OR (('machine'/exp OR machine) AND ('learning'/exp OR learning)) OR 'transfer learning':ab,ti OR 'deep learning':ab,ti OR 'hierarchical learning':ab,ti OR 'ensemble learning':ab,ti OR 'artificial intelligence':ab,ti OR 'prediction model':ab,ti OR 'random forest':ab,ti OR 'neural network':ab,ti OR ann:ab,ti OR 'support vector machine':ab,ti OR svm:ab,ti OR 'gradient boosting machine':ab,ti OR gbm:ab,ti OR nomogram:ab,ti OR xgboost:ab,ti OR adaboost:ab,ti OR 'decision tree':ab,ti OR 'k-nearest neighbor':ab,ti OR 'external validation':ab,ti OR 'risk prediction':ab,ti OR 'risk prediction':ab,ti OR radiomics:ab,ti OR radiomic:ab,ti OR radiogenomic:ab,ti OR 'radiomics based':ab,ti OR texture:ab,ti | 797916 |
| #5 | 'cognitive defect'/exp | 619557 |
| #6 | 'cognitive dysfunction'/exp OR 'cognitive dysfunction' OR (cognitive AND dysfunction) OR 'cognitive dysfunctions':ab,ti OR 'cognitive impairments':ab,ti OR 'cognitive impairment':ab,ti OR 'cognitive disorder':ab,ti OR 'cognitive disorders':ab,ti OR 'mild cognitive impairment':ab,ti OR 'mild cognitive impairments':ab,ti OR 'cognitive decline':ab,ti OR 'cognitive declines':ab,ti OR 'mental deterioration':ab,ti OR 'mental deteriorations':ab,ti OR 'cognitive defect':ab,ti OR 'cognitive defects':ab,ti OR 'cognitive deficit':ab,ti OR 'cognitive disability':ab,ti OR 'cognitive dysfunction':ab,ti OR overinclusion:ab,ti OR 'response interference':ab,ti | 678217 |
| #7 | 'parkinson disease'/exp OR ('parkinson disease'/exp OR 'parkinson disease' OR (parkinson AND ('disease'/exp OR disease)) OR 'primary parkinsonism':ab,ti OR 'parkinsons disease':ab,ti OR 'idiopathic parkinsonism':ab,ti OR 'paralysis agitans':ab,ti OR 'parkinson dementia complex':ab,ti) | 254758 |
| #8 | 'machine learning'/exp OR ('machine learning'/exp OR 'machine learning' OR (('machine'/exp OR machine) AND ('learning'/exp OR learning)) OR 'transfer learning':ab,ti OR 'deep learning':ab,ti OR 'hierarchical learning':ab,ti OR 'ensemble learning':ab,ti OR 'artificial intelligence':ab,ti OR 'prediction model':ab,ti OR 'random forest':ab,ti OR 'neural network':ab,ti OR ann:ab,ti OR 'support vector machine':ab,ti OR svm:ab,ti OR 'gradient boosting machine':ab,ti OR gbm:ab,ti OR nomogram:ab,ti OR xgboost:ab,ti OR adaboost:ab,ti OR 'decision tree':ab,ti OR 'k-nearest neighbor':ab,ti OR 'external validation':ab,ti OR 'risk prediction':ab,ti OR 'risk prediction':ab,ti OR radiomics:ab,ti OR radiomic:ab,ti OR radiogenomic:ab,ti OR 'radiomics based':ab,ti OR texture:ab,ti) | 749916 |
| #9 | 'cognitive defect'/exp OR ('cognitive dysfunction'/exp OR 'cognitive dysfunction' OR (cognitive AND dysfunction) OR 'cognitive dysfunctions':ab,ti OR 'cognitive impairments':ab,ti OR 'cognitive impairment':ab,ti OR 'cognitive disorder':ab,ti OR 'cognitive disorders':ab,ti OR 'mild cognitive impairment':ab,ti OR 'mild cognitive impairments':ab,ti OR 'cognitive decline':ab,ti OR 'cognitive declines':ab,ti OR 'mental deterioration':ab,ti OR 'mental deteriorations':ab,ti OR 'cognitive defect':ab,ti OR 'cognitive defects':ab,ti OR 'cognitive deficit':ab,ti OR 'cognitive disability':ab,ti OR 'cognitive dysfunction':ab,ti OR overinclusion:ab,ti OR 'response interference':ab,ti) | 677217 |
| #10 | ('parkinson disease'/exp OR ('parkinson disease'/exp OR 'parkinson disease' OR (parkinson AND ('disease'/exp OR disease)) OR 'primary parkinsonism':ab,ti OR 'parkinsons disease':ab,ti OR 'idiopathic parkinsonism':ab,ti OR 'paralysis agitans':ab,ti OR 'parkinson dementia complex':ab,ti)) AND ('machine learning'/exp OR ('machine learning'/exp OR 'machine learning' OR (('machine'/exp OR machine) AND ('learning'/exp OR learning)) OR 'transfer learning':ab,ti OR 'deep learning':ab,ti OR 'hierarchical learning':ab,ti OR 'ensemble learning':ab,ti OR 'artificial intelligence':ab,ti OR 'prediction model':ab,ti OR 'random forest':ab,ti OR 'neural network':ab,ti OR ann:ab,ti OR 'support vector machine':ab,ti OR svm:ab,ti OR 'gradient boosting machine':ab,ti OR gbm:ab,ti OR nomogram:ab,ti OR xgboost:ab,ti OR adaboost:ab,ti OR 'decision tree':ab,ti OR 'k-nearest neighbor':ab,ti OR 'external validation':ab,ti OR 'risk prediction':ab,ti OR 'risk prediction':ab,ti OR radiomics:ab,ti OR radiomic:ab,ti OR radiogenomic:ab,ti OR 'radiomics based':ab,ti OR texture:ab,ti)) AND ('cognitive defect'/exp OR ('cognitive dysfunction'/exp OR 'cognitive dysfunction' OR (cognitive AND dysfunction) OR 'cognitive dysfunctions':ab,ti OR 'cognitive impairments':ab,ti OR 'cognitive impairment':ab,ti OR 'cognitive disorder':ab,ti OR 'cognitive disorders':ab,ti OR 'mild cognitive impairment':ab,ti OR 'mild cognitive impairments':ab,ti OR 'cognitive decline':ab,ti OR 'cognitive declines':ab,ti OR 'mental deterioration':ab,ti OR 'mental deteriorations':ab,ti OR 'cognitive defect':ab,ti OR 'cognitive defects':ab,ti OR 'cognitive deficit':ab,ti OR 'cognitive disability':ab,ti OR 'cognitive dysfunction':ab,ti OR overinclusion:ab,ti OR 'response interference':ab,ti)) | 2374 |

**4.Web of science**

| Search number | Query | Results |
| --- | --- | --- |
| #1 | Parkinson Disease (topical) OR Parkinson dementia complex (topical) OR Parkinson's Disease (topical) OR Primary Parkinsonism (topical) OR Paralysis Agitans (topical) OR Parkinsons disease (topical) OR idiopathic parkinsonism (topical) OR paralysis agitans (topical) | 267395 |
| #2 | machine learning (topical) OR Transfer Learning (topical) OR Deep learning (topical) OR Hierarchical Learning (topical) OR Ensemble Learning (topical) OR artificial intelligence (topical) OR Prediction model (topical) OR random forest (topical) OR neural network (topical) OR ANN (topical) OR Support vector machine (topical) OR SVM (topical) OR Gradient Boosting Machine (topical) OR GBM (topical) OR Nomogram (topical) OR XGboost (topical) OR Adaboost (topical) OR Decision tree (topical) OR K-Nearest Neighbor (topical) OR External validation (topical) OR Risk Prediction (topical) OR Risk-Prediction (topical) OR Radiomics (topical) OR radiomic (topical) OR radiogenomic (topical) OR radiomics-based (topical) OR Texture (topical) | 3624298 |
| #3 | Cognitive Dysfunction (topical) OR Cognitive Dysfunctions (topical) OR Cognitive Impairments (topical) OR Cognitive Impairment (topical) OR Cognitive Disorder (topical) OR Cognitive Disorders (topical) OR Mild Cognitive Impairment (topical) OR Mild Cognitive Impairments (topical) OR Cognitive Decline (topical) OR Cognitive Declines (topical) OR Mental Deterioration (topical) OR Mental Deteriorations (topical) OR cognitive defect (topical) OR cognitive defects (topical) OR cognitive deficit (topical) OR cognitive disability (topical) OR cognitive dysfunction (topical) OR overinclusion (topical) OR response interference (topical) | 705348 |
| #4 | #1 AND #2 AND #3 | 2654 |

**
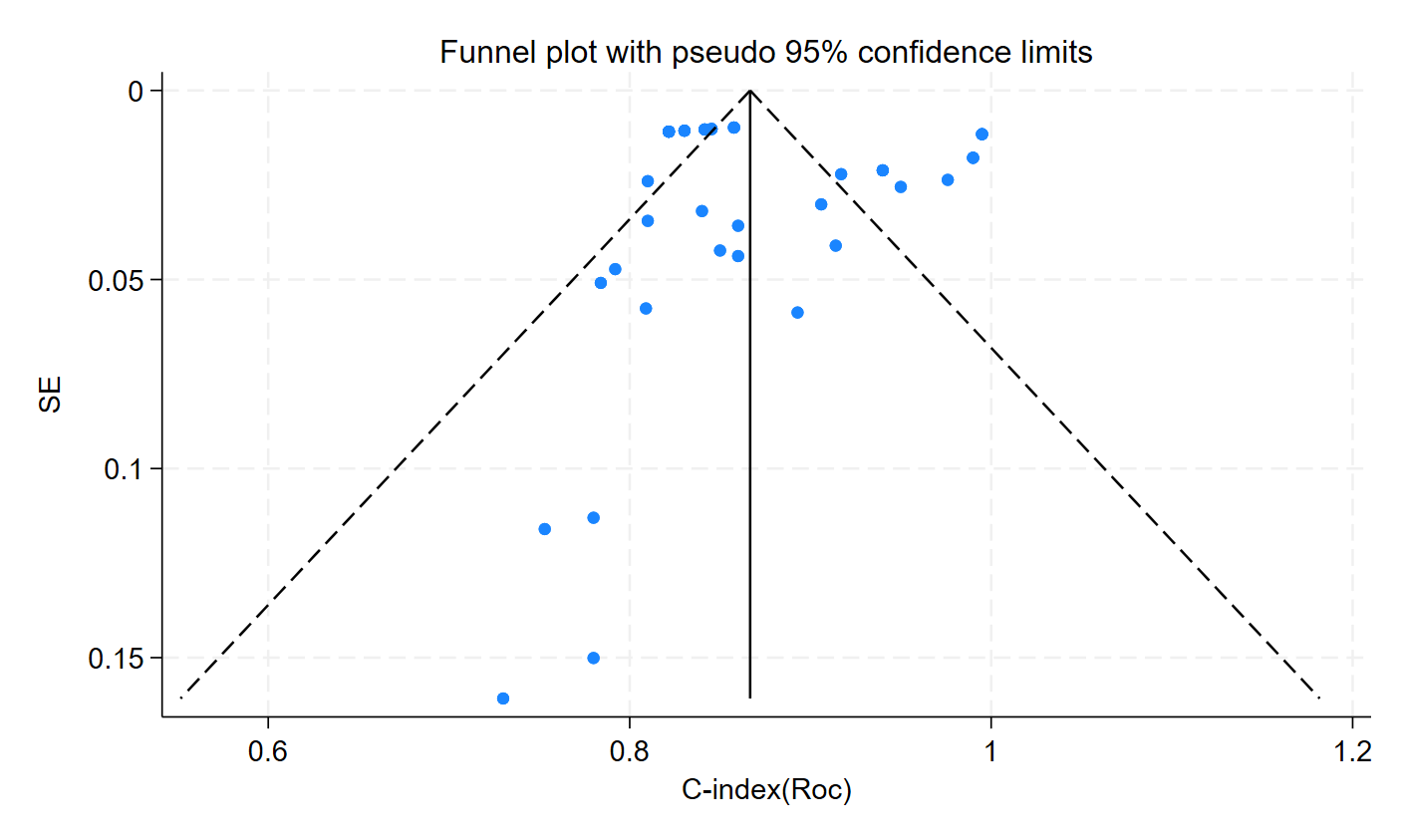
Supplementary Material 3.** Funnel plot of the diagnostic models in the training set

**
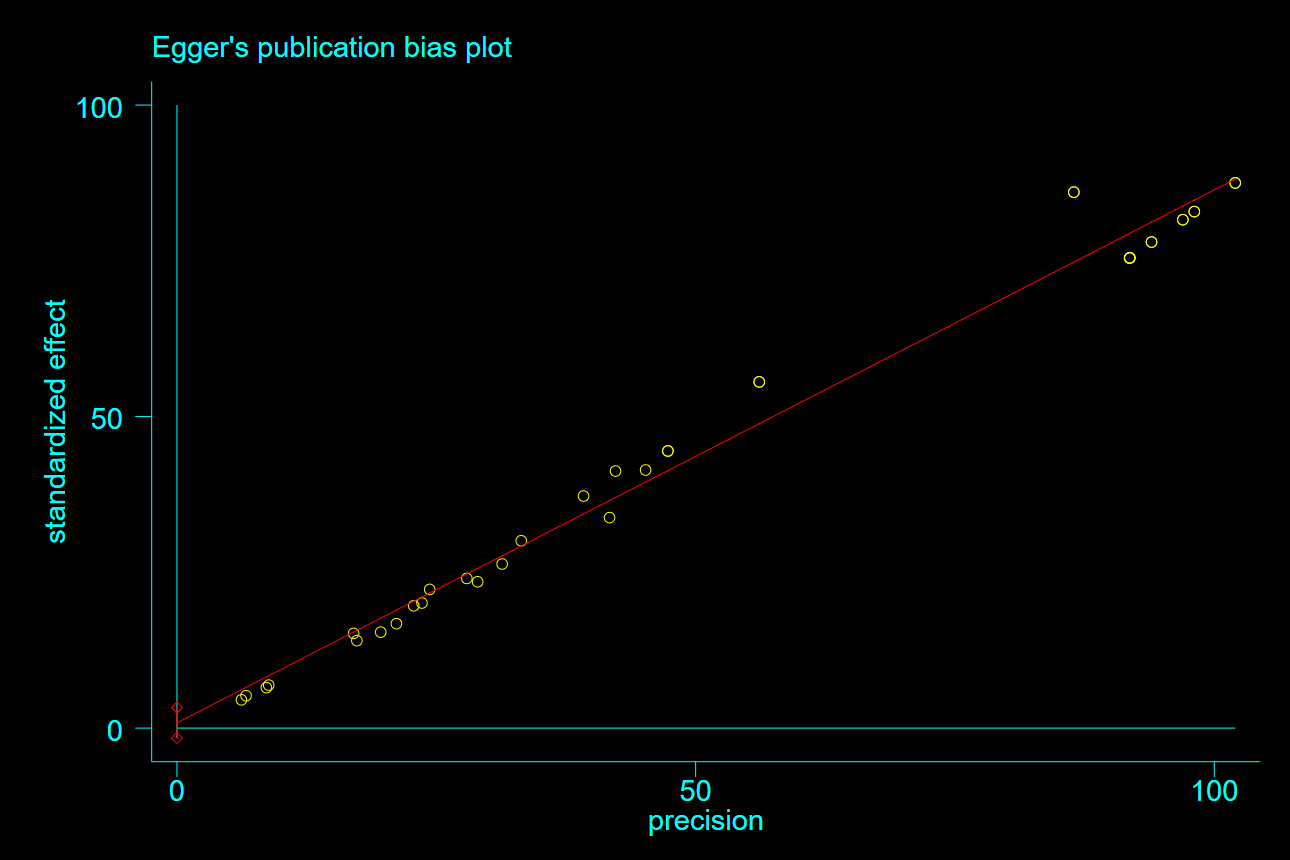
Supplementary Material 4.** Egger’s test for the diagnostic models in the training set

**
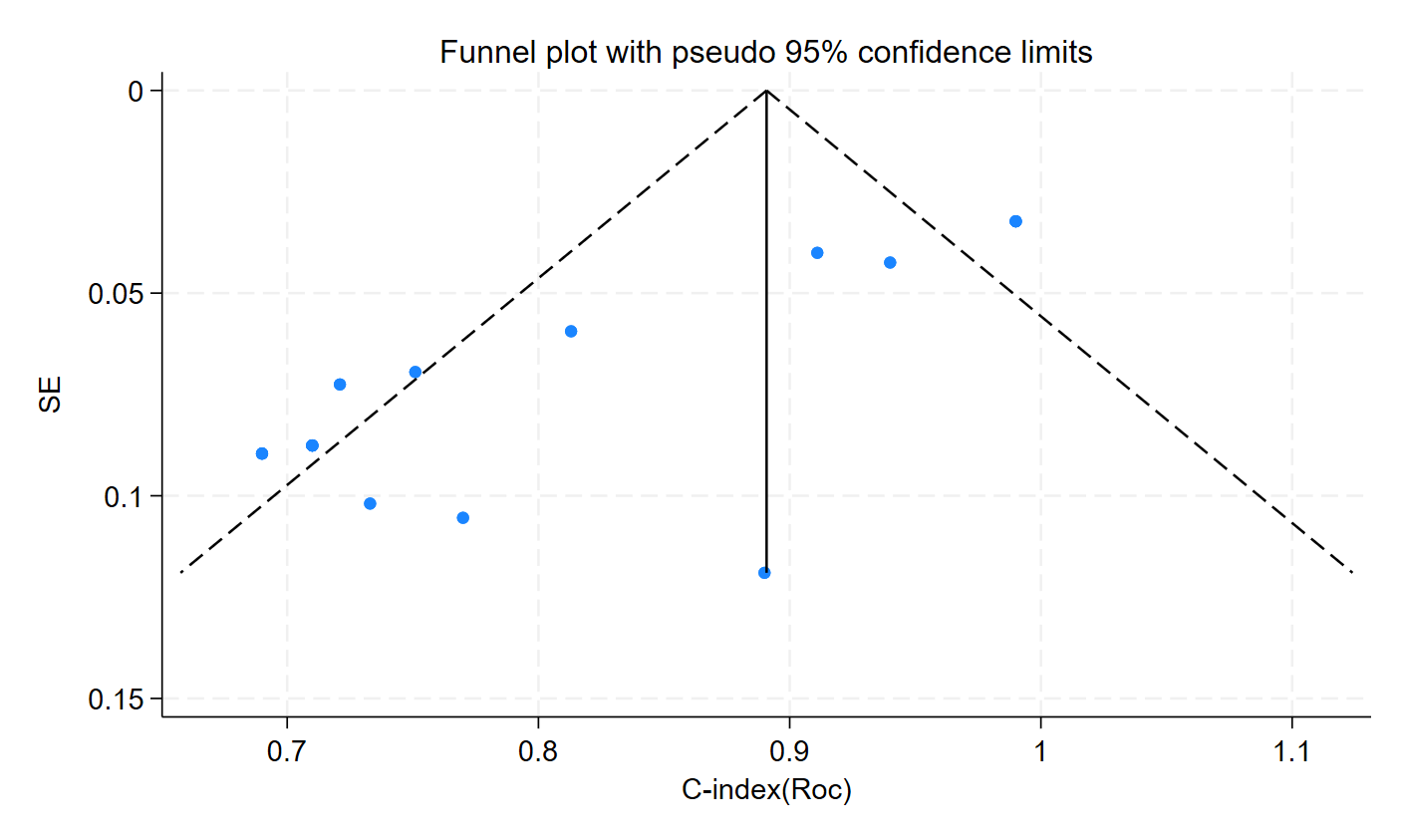
Supplementary Material 5.** Funnel plot of the diagnostic models in the validation set

**
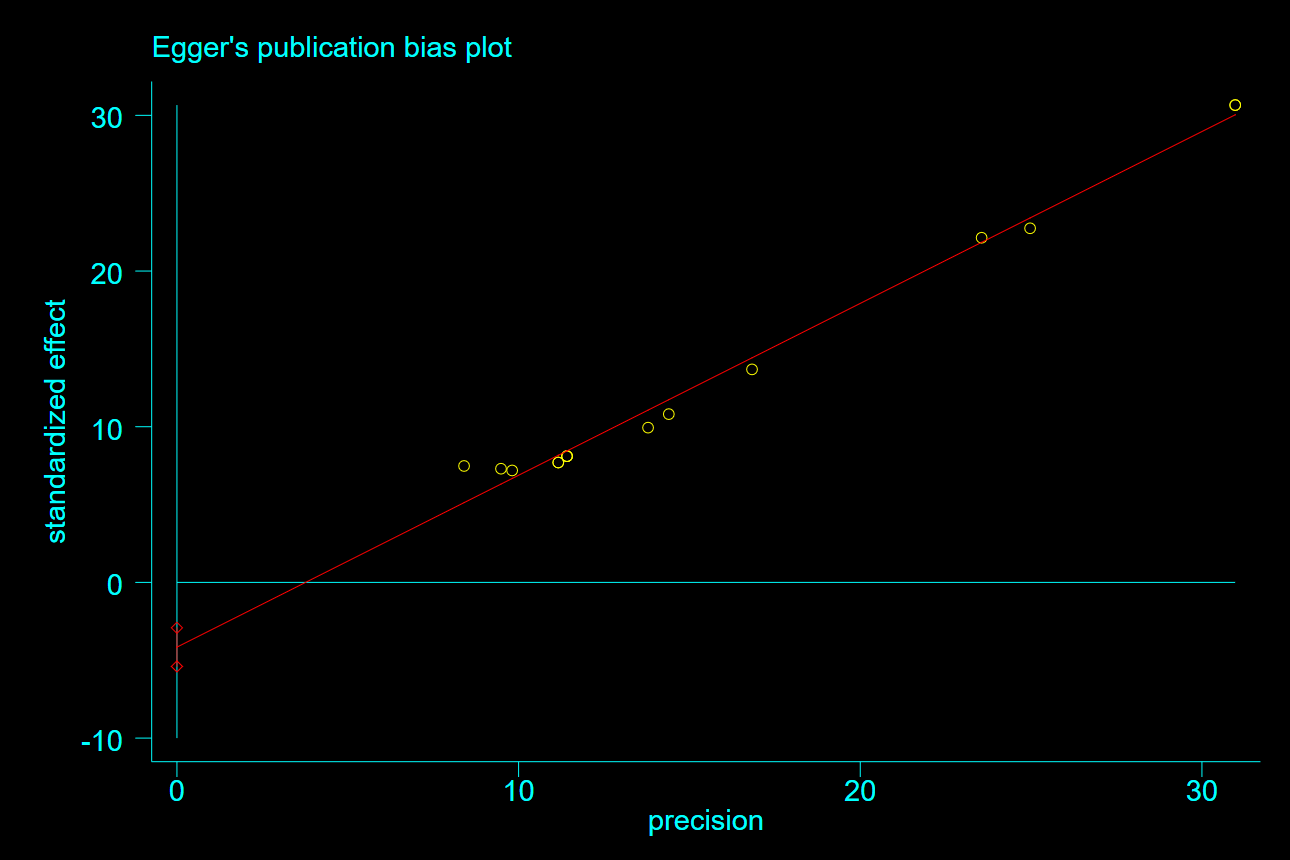
Supplementary Material 6.** Egger’s test for the diagnostic models in the validation set

**
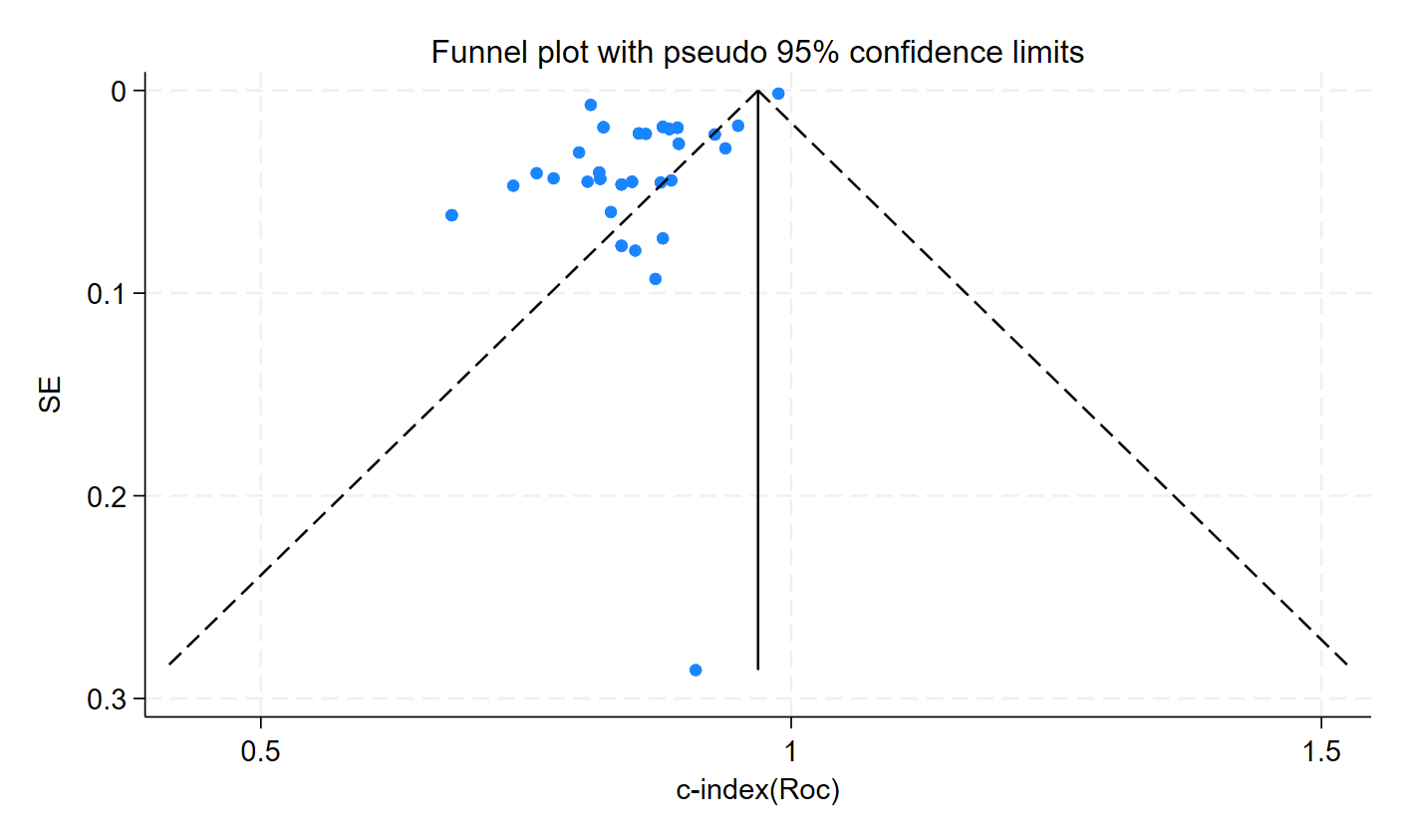
Supplementary Material 7.** Funnel plot of the prediction models in the training set

**
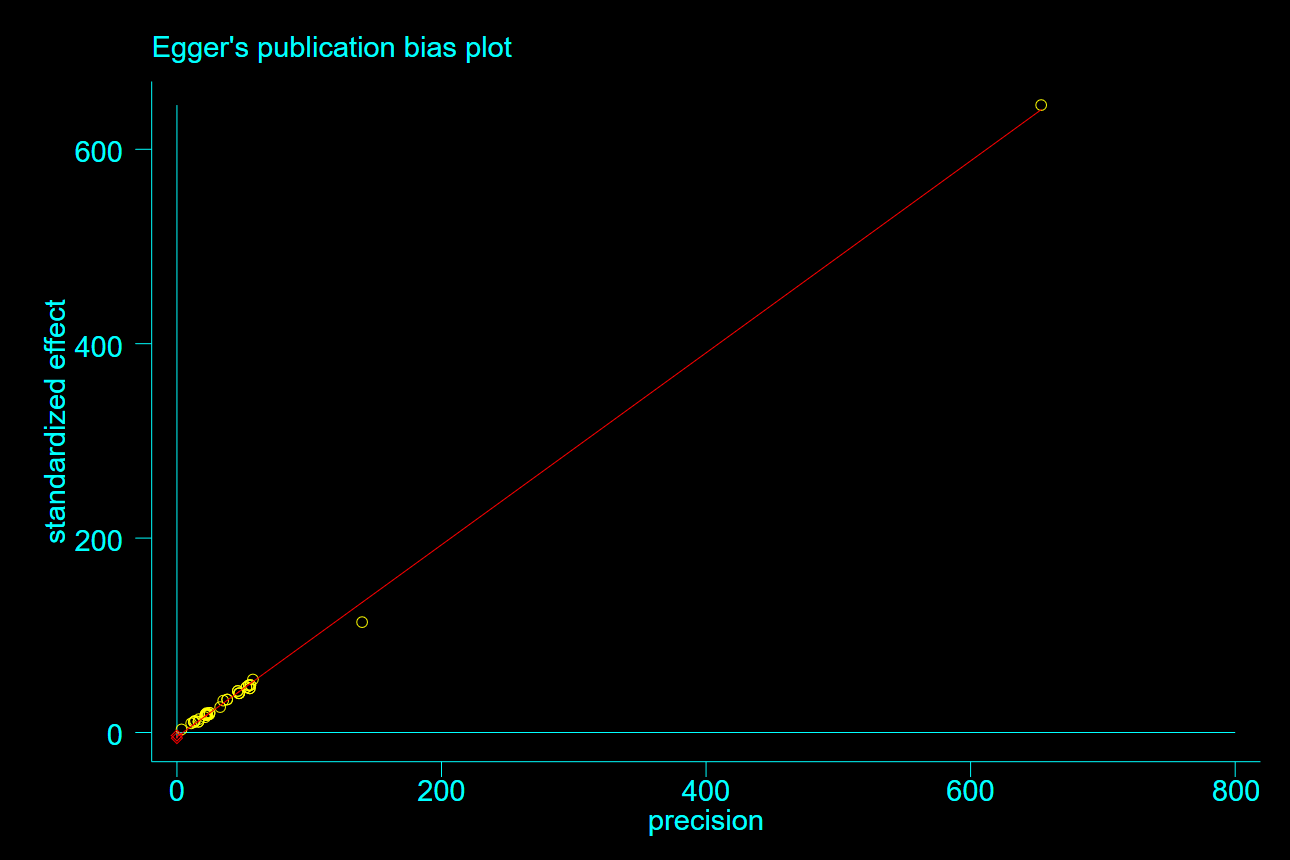
Supplementary Material 8.** Egger’s test for the prediction models in the training set

**
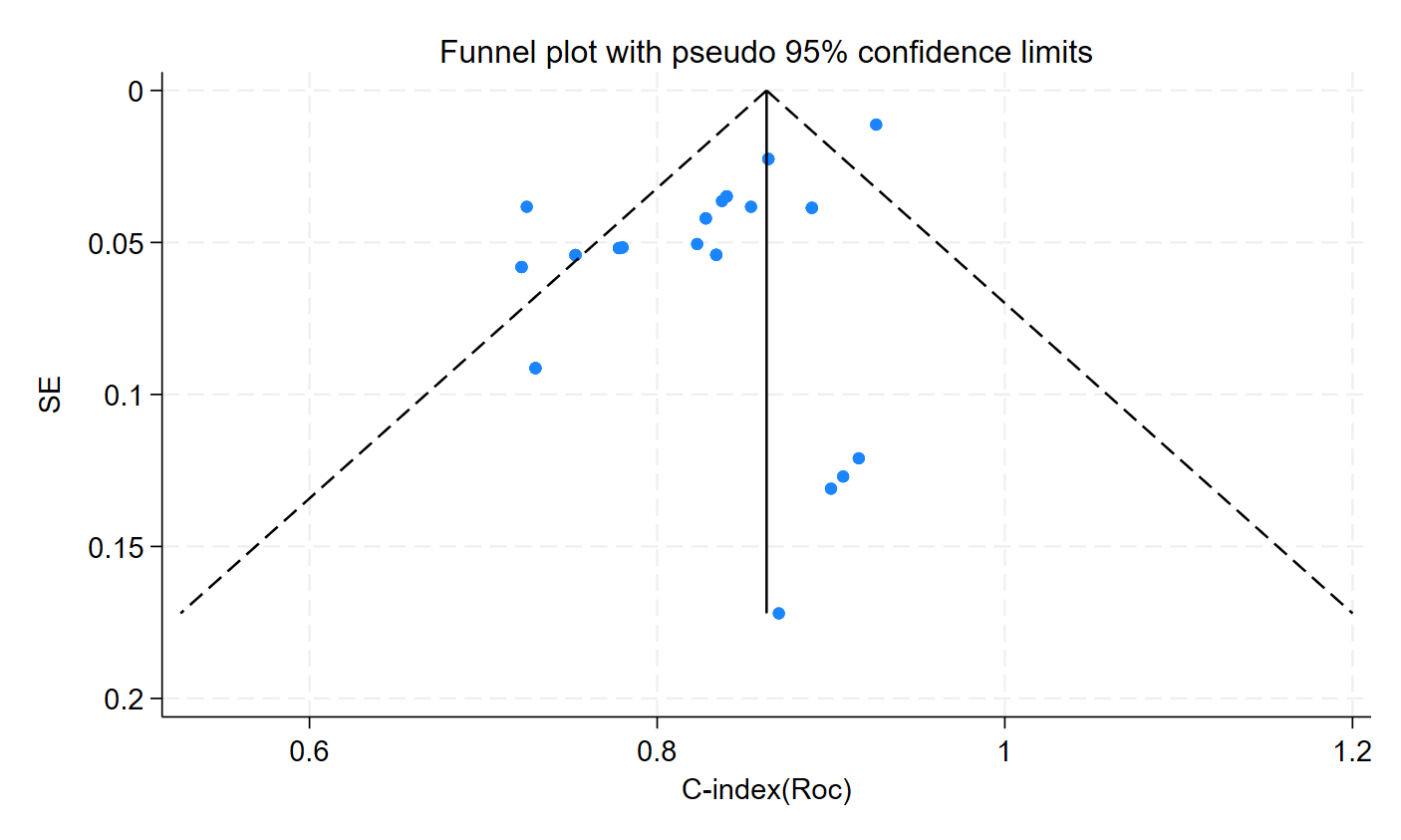
Supplementary Material 9.** Funnel plot of the prediction models in the validation set

**
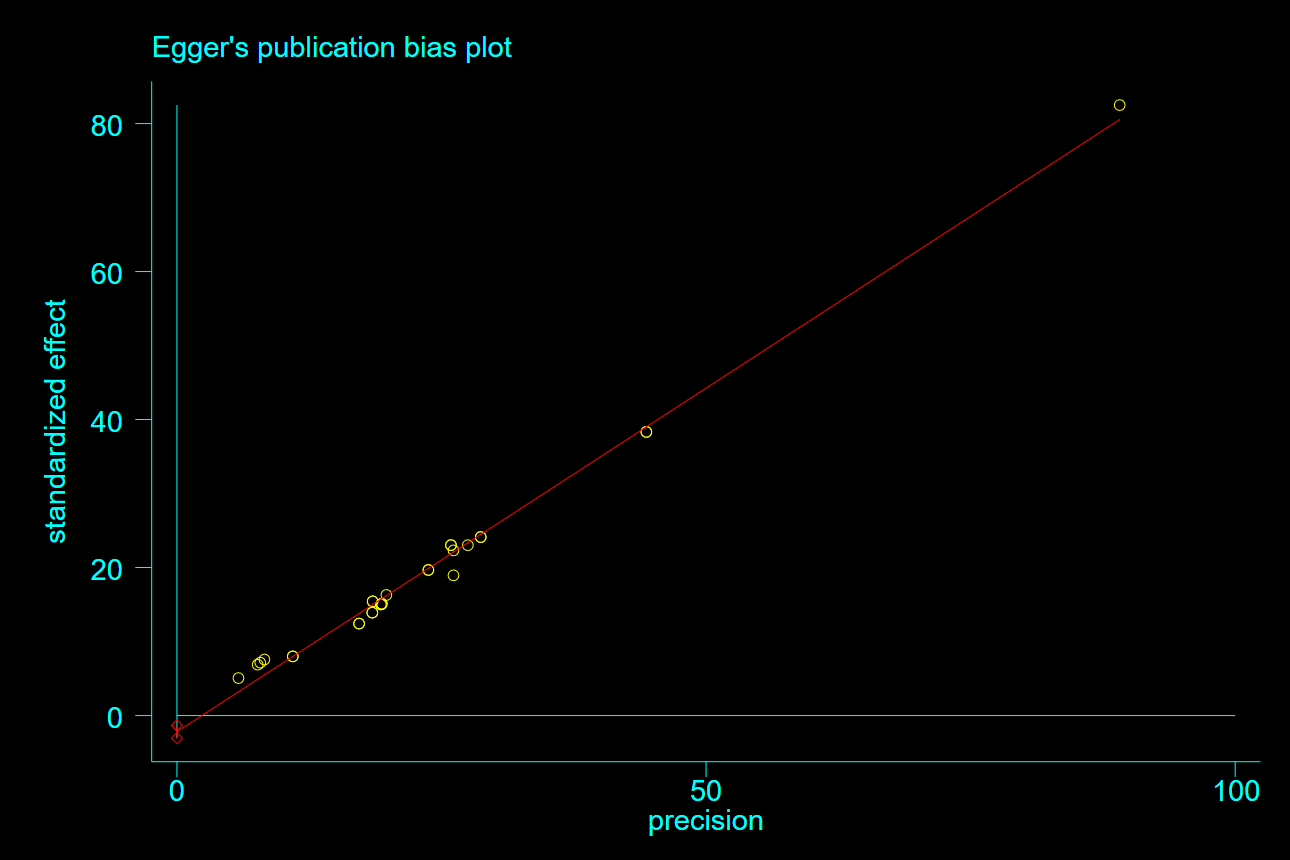
Supplementary Material 10.** Egger’s test for the prediction models in the validation set

**
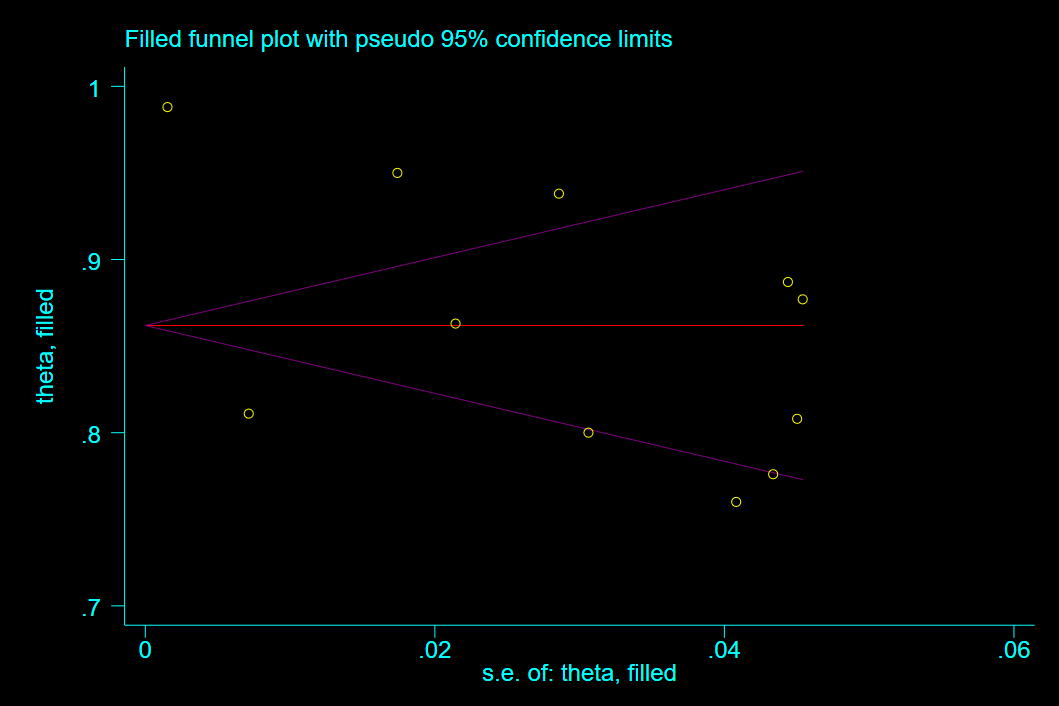
**

**Supplementary Material 11.** Trim-and-fill method for the diagnostic models in the validation set

**
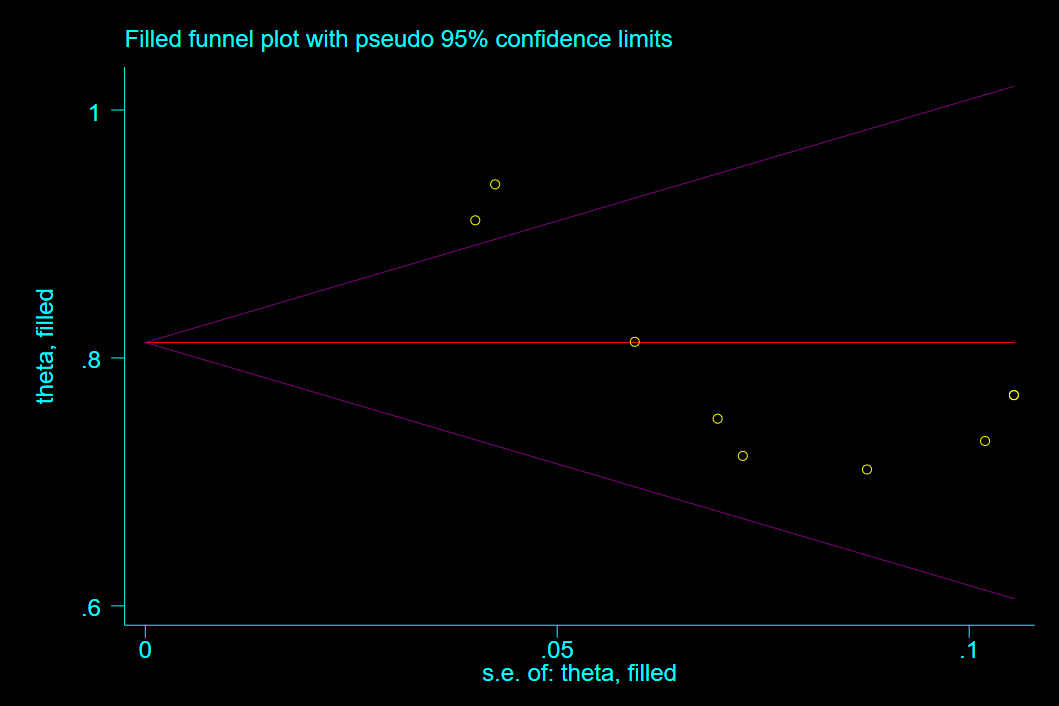
**

**Supplementary Material 12.** Trim-and-fill method for the prediction models in the training set

**
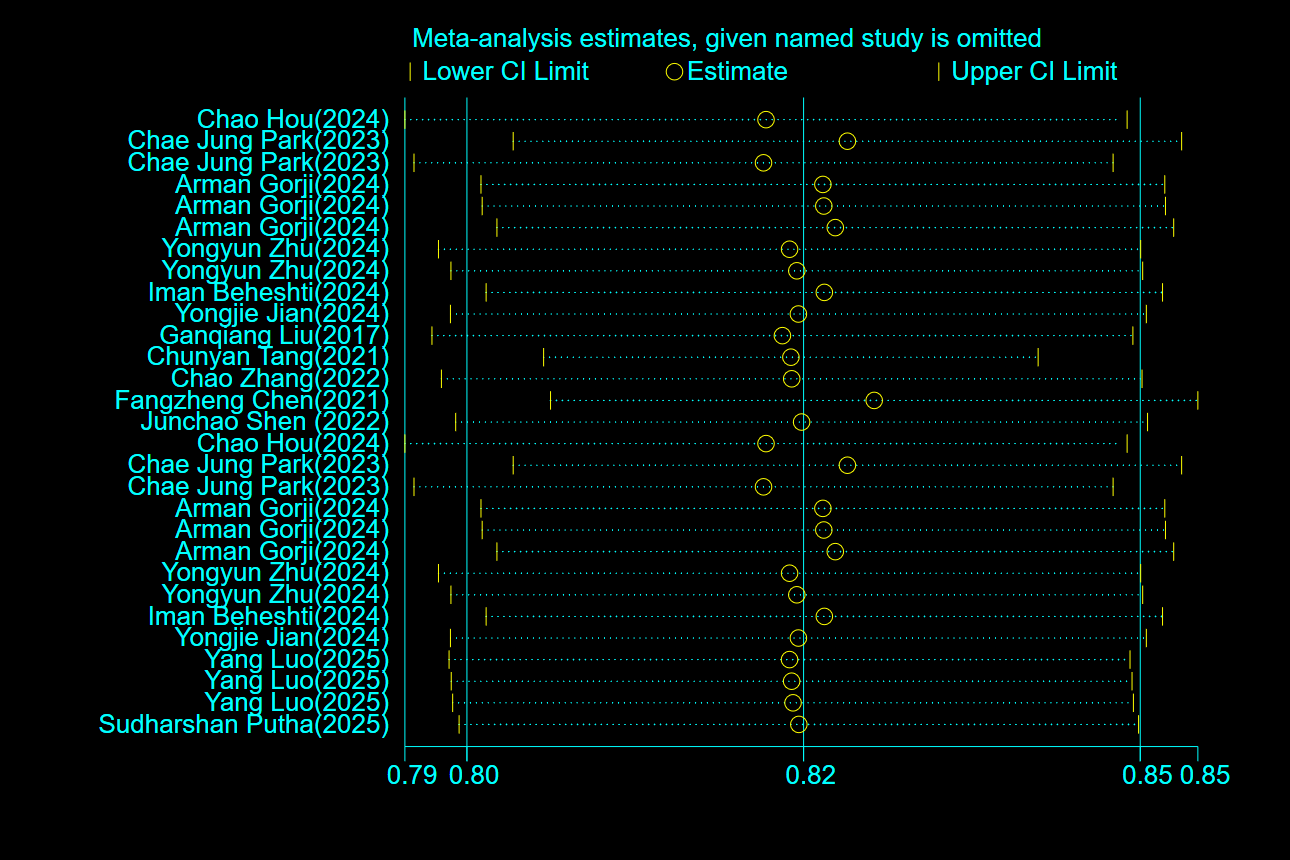
**

**Supplementary Material 13.** Sensitivity analysis of the diagnostic models in the training set

**
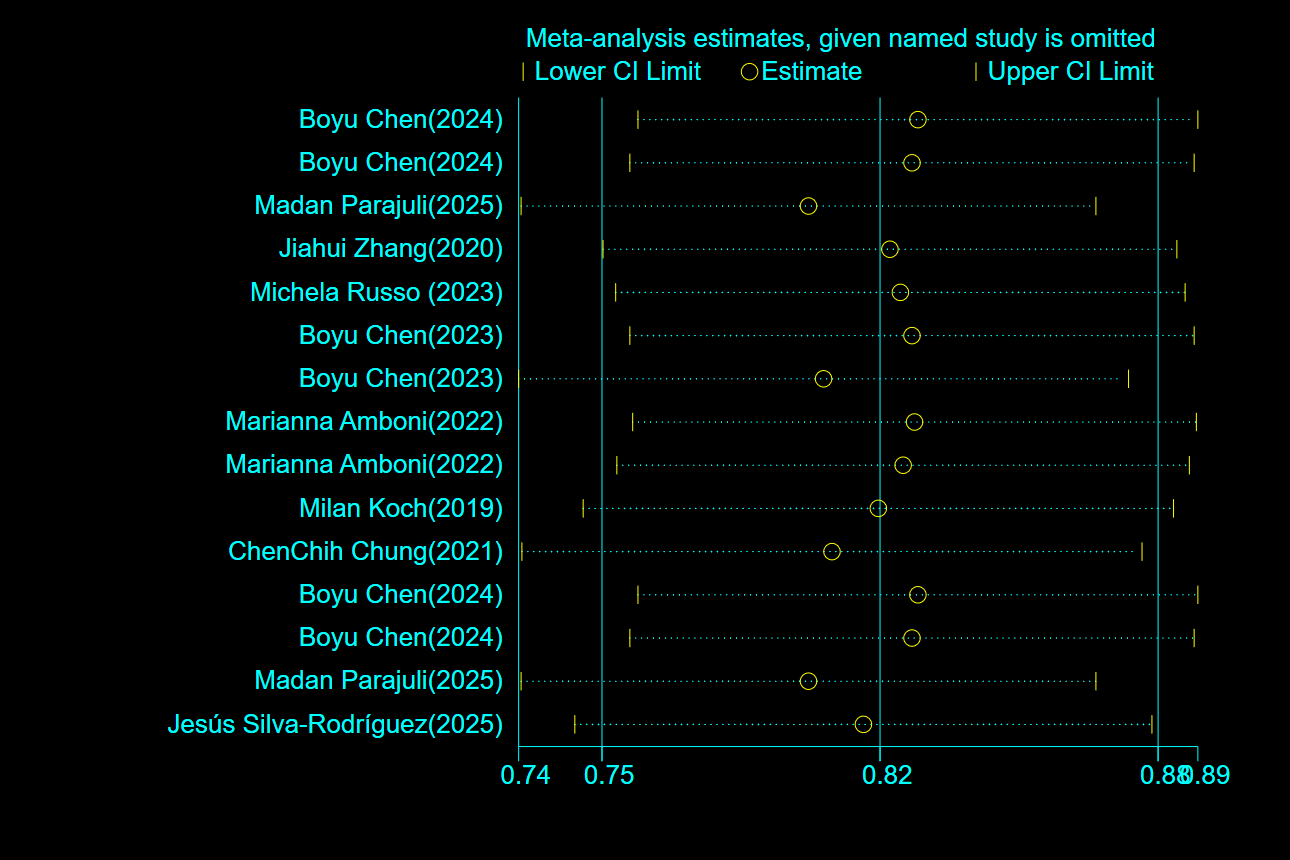
Supplementary Material 14.** Sensitivity analysis of the diagnostic models in the validation set

**
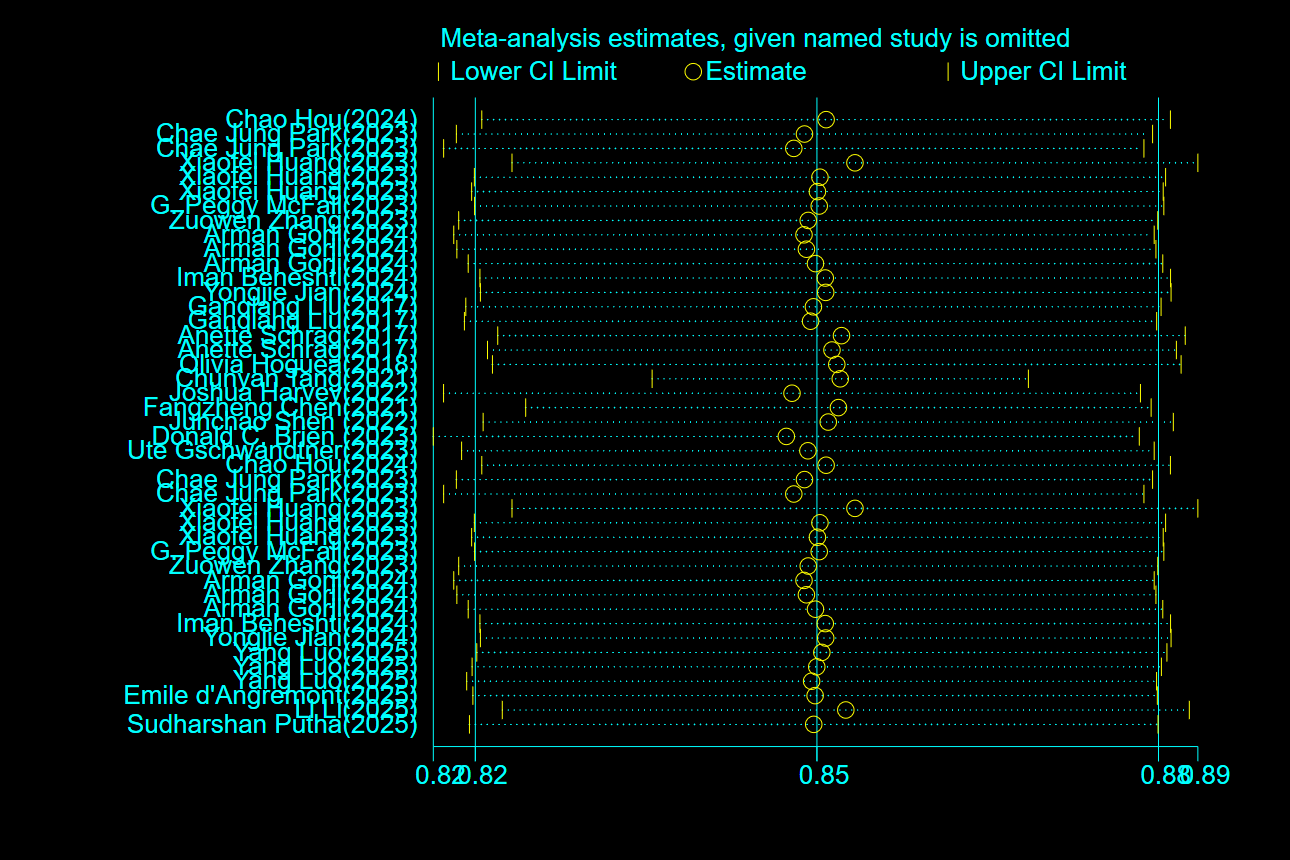
**

**Supplementary Material 15.** Sensitivity analysis of the prediction models in the training set

**
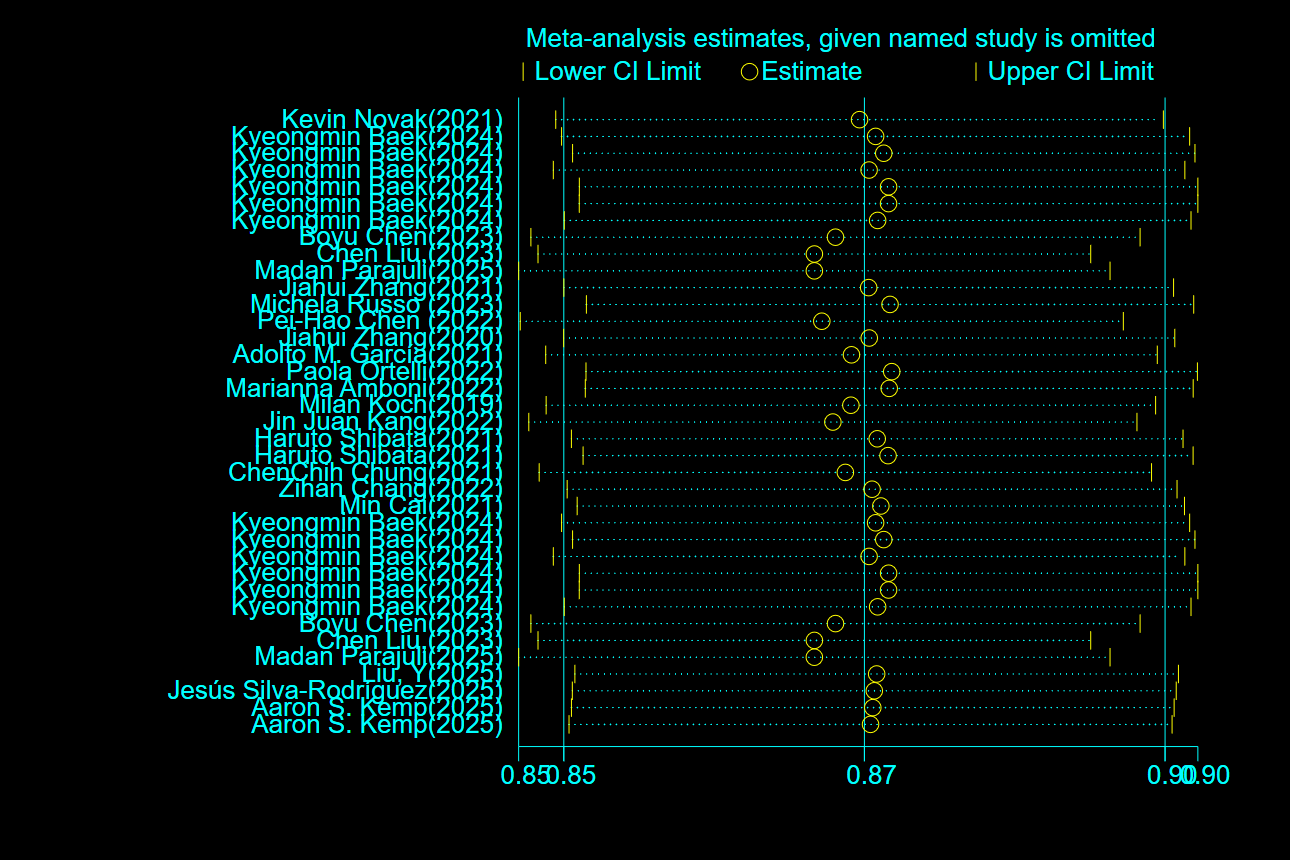
**

**Supplementary Material 16.** Sensitivity analysis of the prediction models in the validation set
